# A comprehensive framework for legal dispute analysis integrating prompt engineering and multi-dimensional knowledge graphs

**DOI:** 10.1038/s41598-025-30306-9

**Published:** 2025-12-18

**Authors:** Mingda Zhang, Na Zhao, Jianglong Qin, Qing Xu, Kaiwen Pan, Ting Luo

**Affiliations:** 1 School of Software, Yunnan University, Kunming, 650500 China; 2 Yunnan Key Laboratory of Software Engineering, Kunming, 650500 China; 3 School of Law, Yunnan University, Kunming, 650091 China

**Keywords:** Legal dispute analysis, Prompt engineering, Multi-dimensional knowledge graph, Knowledge enhancement, Analysis workflow, Mathematics and computing, Science, technology and society

## Abstract

Legal dispute analysis is crucial for intelligent legal assistance systems. However, current Large Language Models (LLMs) face challenges in understanding complex legal concepts, maintaining reasoning consistency, and accurately citing legal sources. This study presents a framework combining prompt engineering with multi-dimensional knowledge graphs to improve LLM capabilities for legal dispute analysis. The framework comprises a three-stage hierarchical prompt structure (task definition, knowledge background, reasoning guidance) and a three-layer knowledge graph (legal classification ontology layer, representation layer, instance layer). Additionally, four supporting methods enable legal concept retrieval: direct code matching, semantic vector similarity, ontology path reasoning, and professional terminology matching. Systematic testing on 500 test samples integrated from six internationally recognized legal AI benchmark datasets demonstrates performance improvements for mainstream models: F1 score increased from 0.356 to 0.714, BLEU-4 reached 0.451, ROUGE-L F1 improved from 0.34 to 0.71, and legal professional content quality scores increased by 18-20 points (on a 100-point scale). This framework provides a technical approach for legal analysis, contributing to the advancement of intelligent legal assistance systems.

## Introduction

Legal dispute analysis, as a core cognitive task in judicial practice, requires legal professionals to systematically parse conflicting claims, evaluate chains of evidence, and provide judicial solutions. Industry surveys indicate that legal professionals spend 30-50% of their working time researching applicable legal provisions and precedent cases^1^. This situation highlights both the complexity of legal analytical work and the need for intelligent assistance technologies. Recently released evaluation benchmarks such as LegalBench demonstrate the multi-dimensional challenges of legal reasoning tasks^2^, providing a standardized framework for systematic evaluation of legal Artificial Intelligence (AI) capabilities.

The emergence of LLMs has brought new technical pathways for intelligent legal assistance systems. A 2024 Wolters Kluwer survey shows that 76% of legal departments and 68% of law firms have incorporated AI tools into their daily workflows, marking the transition of legal AI from experimental exploration to the application stage. However, this application has also exposed technical challenges: industry research indicates that existing generative AI systems may have high rates of “hallucination” in legal queries^3^. Multiple jurisdictions, including California, have seen cases of lawyer sanctions due to AI-generated false case citations, prompting judicial institutions to advance AI regulatory frameworks. These practical problems stem from structural limitations of LLMs: their parameter scale exhibits diminishing marginal returns with performance improvement—Wu et al.’s^4^ parameter scale experiments reveal that when models expand from 1 billion to 10 billion parameters, computational resource requirements grow exponentially while performance improvements follow a logarithmic curve, creating a “scale dilemma.” Meanwhile, existing legal language models have room for improvement in deep representation of legal knowledge, understanding of professional concepts, and cross-jurisdictional reasoning^5^.

To address these challenges, this study proposes a framework for legal dispute analysis that integrates prompt engineering with multi-dimensional knowledge graphs. The framework is based on the technical concept of “selective knowledge node retrieval,” which maintains reasoning performance while reducing computational costs by locating relevant legal concepts rather than loading the entire knowledge base^1^. Compared with existing legal AI systems, this framework constructs a knowledge enhancement ecosystem including legal concept retrieval, multi-level knowledge representation, and professional reasoning path prompting^6^. This design helps improve LLM capabilities in legal norm application and case analysis. Research shows that LLMs enhanced with legal professional feedback can improve legal reasoning abilities^7^, but knowledge expression optimization and reasoning path design remain challenges. From the perspective of legal practice regulation, legal AI systems need to balance technological innovation and professional standardization, ensuring outputs meet legal professionalism and ethical requirements.

Based on the core challenges identified in legal dispute analysis, this study provides two technical contributions through the integration of prompt engineering and multi-dimensional knowledge graphs:

**Legal three-stage prompt engineering framework:** We design a hierarchical prompt architecture composed of task definition, knowledge background, and reasoning guidance, achieving the transformation from static templates to adaptive enhancement through dynamic optimization mechanisms^8,9^.

**Multi-dimensional knowledge graph and multi-strategy retrieval system:** We construct a three-layer architecture knowledge graph with complementary retrieval strategies, enabling dynamic retrieval of legal concepts and management of knowledge timeliness^10,11^.

## Related work

### Applications and challenges of large language models in legal dispute analysis

Recent advances in LLMs have catalyzed progress in legal AI applications. Professional legal AI systems such as Harvey AI and Casetext CoCounsel have demonstrated the viability of combining specialized legal knowledge with large-scale models through professional tuning^1,12^. Retrieval-Augmented Generation (RAG) technology has emerged as a promising pathway, with platforms adopting this approach showing improved accuracy in independent tests^13^. The latest developments in 2025 advance this trajectory: unified retrieval frameworks enable cross-task legal applications^14^, comprehensive benchmarks systematically evaluate agent performance^15^, and step-by-step verification mechanisms enhance reasoning accuracy^16^.

However, structural limitations persist despite these advances. The specialized nature of legal knowledge, coupled with terminology precision requirements and jurisdictional variations, creates knowledge gaps that general-purpose training cannot fully address^5^. High-profile incidents in 2024, including attorney sanctions for AI-generated false case citations in California courts, underscore the severity of hallucination problems in legal queries^3^. Three core challenges impede progress: first, data scarcity due to attorney-client privilege restrictions limits access to high-quality legal training data; second, rapid iteration of legal knowledge through amendments and judicial interpretations demands timely model updates; third, the multi-layered complexity of legal reasoning requires integration of principles, provisions, precedents, and specific facts^17–19^. This study addresses these challenges through the integration of prompt engineering with multi-dimensional knowledge graphs, implementing selective knowledge node retrieval to balance reasoning performance with computational efficiency.

### Development of prompt engineering in professional domains

Prompt engineering has evolved as a non-invasive optimization technique for LLMs, enabling task adaptation without parameter modification. Chain-of-Thought prompting pioneered the approach of enhancing complex reasoning through explicit intermediate steps^8,20^. Building on this foundation, domain-specific applications have emerged: multi-stage frameworks optimize legal document generation^21^, while structured prompts integrating professional knowledge improve judgment prediction accuracy^22^. Recent research in 2024-2025 reveals a paradigm shift from static templates to dynamic optimization strategies, with principled instructions and follow-up prompts yielding performance improvements of 16% and 9.2% respectively^23,24^. Parameter-efficient fine-tuning techniques such as LoRA and Prefix-tuning enable domain adaptation while maintaining frozen model parameters.

Despite these advances, applicability gaps remain in legal contexts. The structured nature of legal reasoning demands adherence to professional argumentation frameworks such as IRAC (Issue, Rule, Application, Conclusion), terminology precision requires accurate disambiguation of legal meanings, and citation standardization necessitates compliance with jurisdiction-specific norms^25^. More critically, existing research predominantly addresses isolated legal tasks rather than comprehensive dispute analysis, lacking systematic integration with multi-dimensional knowledge structures^26^. Traditional flat prompt architectures face difficulties with complex legal scenarios requiring synthesis of multiple concepts and multi-level reasoning. The legal three-stage prompt engineering framework proposed in this study addresses these limitations through hierarchical architecture integrating task definition, knowledge background, and reasoning guidance. Dynamic task identification algorithms map queries to professional templates, legal reasoning path templates provide domain-specific guidance, and adaptive optimization mechanisms transform prompt engineering from “one-time generation” to “iterative refinement” through multi-dimensional quality assessment feedback.

### Current status and challenges of multi-dimensional knowledge graphs

Knowledge graphs constitute the foundational infrastructure for legal knowledge representation and reasoning. Recent progress has transitioned construction methodologies from manual annotation to automation: joint knowledge enhancement models embedding prior knowledge into LLMs achieve automated construction of legal knowledge graphs with performance improvements in entity extraction and relationship identification^27,28^. The fusion of knowledge graphs with RAG technology has become a development direction, with hybrid retrieval systems combining vector databases and knowledge graphs demonstrating enhanced legal information retrieval accuracy^29^. Cross-lingual and temporal adaptation capabilities have also advanced through specialized techniques: cross-lingual paragraph retrieval methods^30^ and progressive modular adapters^31^ enable multi-jurisdictional applications, while dynamic mixture-of-experts mechanisms enhance temporal generalization^32^.

Despite these advances, challenges persist in knowledge representation granularity, timeliness maintenance, and complex relationship expression. Technical obstacles concentrate in two areas: first, knowledge acquisition and representation face high specialization demands—hierarchical relationships, citation networks, and applicability constraints among legal concepts resist full capture by traditional knowledge graphs^33^; second, timeliness requirements necessitate rapid incorporation of legal amendments, judicial interpretations, and landmark cases. Additionally, most existing legal knowledge graphs focus on single jurisdictions or specific domains with limited cross-domain integration capabilities, dynamic update mechanisms remain imperfect for reflecting rapid legal environment changes, and collaborative optimization pathways between knowledge graphs and prompt engineering remain underexplored^34^. Addressing these limitations, this study designs a multi-dimensional knowledge graph with three-layer architecture encompassing legal classification ontology, legal representation, and legal instance layers, achieving coverage from abstract concepts to concrete applications^35^. Four complementary retrieval strategies—direct legal code matching, semantic vector similarity, ontology path reasoning, and professional terminology matching—enable dynamic concept retrieval. Furthermore, unified retrieval interfaces covering authoritative legal data sources ensure accuracy and timeliness through jurisdictional identification, timeliness marking, and change tracking mechanisms, providing knowledge support for legal dispute analysis^34^.

## Key technical design and implementation of the legal dispute analysis framework

**Fig. 1:**
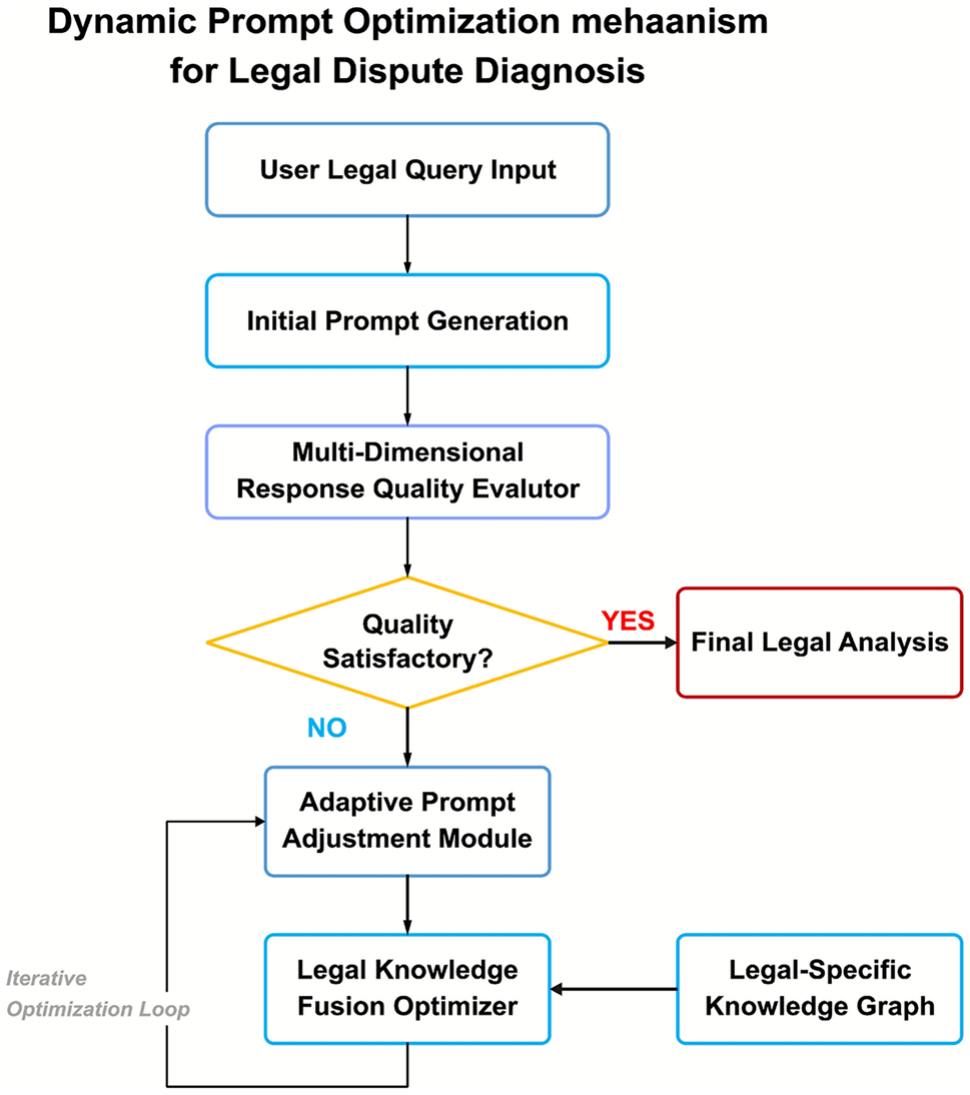
Overall architecture of the legal dispute analysis framework.

The legal dispute analysis framework constructs a legal dispute analysis technical ecosystem through the integration of legal three-stage prompt engineering and multi-dimensional knowledge graphs as two core technologies, achieving full-process enhancement from legal concept identification and knowledge acquisition to reasoning guidance. As shown in Fig. 1, the multi-dimensional knowledge graph (left side) and three-stage prompt engineering (right side) constitute the dual core of the system architecture, presenting a dual-module collaborative design concept where the prompt engineering module is responsible for legal reasoning guidance and the knowledge graph module provides legal knowledge support. When users input legal dispute queries, the system first identifies key legal concepts through multi-granularity concept retrieval components while activating knowledge graph query modules to obtain relevant legal knowledge, which is then integrated by the three-stage prompt engineering module to generate structured prompts containing task definition, knowledge background, and reasoning guidance, directing LLMs to generate professional legal responses. The figure displays the data flow transmission paths between components and the logical connections between functional modules, reflecting how this dual-core design achieves the integration of knowledge retrieval and reasoning guidance while ensuring system robustness and adaptability when facing complex legal issues.

### Legal three-stage prompt engineering framework

Prompt engineering, as a key technology for guiding LLM behavior, achieves task adaptation and performance improvement by designing input instructions while keeping model parameters unchanged. Unlike model fine-tuning methods that require large amounts of annotated data and computational resources, prompt engineering adopts a non-invasive design, shaping model outputs only by optimizing the text structure and content of the input layer, making it suitable for Application Programming Interface (API) service scenarios where model weights cannot be accessed and application development environments requiring rapid iteration. However, traditional prompt engineering methods suffer from issues such as single structure, lack of professional legal thinking paths, and insufficient reasoning depth. The three-stage prompt engineering framework proposed in this study addresses the limitations of flat structures, designing a hierarchical architecture containing three levels: task definition, knowledge background, and reasoning guidance. Through structured prompt design and external knowledge injection, it enhances legal reasoning capabilities under the condition of frozen model parameters. The effectiveness of structured legal knowledge understanding frameworks has been validated in multiple studies^9,23^, providing empirical support for the hierarchical prompt structure design of this study.

#### Task definition and precise role positioning mechanism

The legal task identification matching algorithm achieves mapping from queries to professional task templates through a multi-dimensional matching mechanism. The core of this algorithm lies in considering multiple feature dimensions such as legal domain categories, problem nature, and involved legal provisions, calculating the matching degree between queries and task templates through an improved BM25F-style weighting mechanism. To avoid excessive accumulation of constant terms caused by over-tokenization, this study places the BM25+ constant term at the field aggregation layer. The calculation formula is as follows:

1 $$\begin{aligned} M(Q, T_i) = \sum _{j=1}^{n} w_j \left[ \sum _{t\in Q\cap T_i} \text {IDF}_j(t)\cdot \frac{f_j(t,T_i)\,(k_{1j}+1)}{f_j(t,T_i)+k_{1j}\left( 1-b_j+b_j\frac{|T_i|}{\text {avgdl}_j}\right) }\right] + \sum _{j=1}^{n} w_j\cdot \delta _j\cdot \mathbb {I}[|Q\cap T_i|>0] \end{aligned}$$

Here, *Q* represents the user’s legal query text, $T_i$ represents the *i*-th predefined legal task template, and *n* represents the number of feature dimensions (including legal domain categories, problem nature, involved legal provisions, etc.). The first term is the term matching score based on BM25, and the second term is the field-level constant gain term, where $\mathbb {I}[|Q\cap T_i|>0]$ is an indicator function that adds the $\delta _j$ gain only when that feature dimension has a match, avoiding excessive accumulation of constant terms due to term fragmentation.

$w_j$ represents the importance weight of the *j*-th feature dimension, with initial values determined through the Delphi method followed by grid search fine-tuning on annotated samples, satisfying the weight normalization condition $\sum _{j=1}^{n} w_j = 1$. The optimized weights in this experiment are: legal domain category $w_1=0.35$, problem nature $w_2=0.28$, involved provisions $w_3=0.22$, other features $w_4=0.15$. $f_j(t,T_i)$ represents the term frequency of term *t* in the *j*-th dimension feature of template $T_i$, $|T_i|$ represents template length, and $\text {avgdl}_j$ represents the average document length of the *j*-th dimension feature. $k_{1j}, b_j, \delta _j$ are field-level parameters set according to field types (main text, classification, encoding) to adapt to the heterogeneous characteristics of legal texts, where main text fields adopt BM25+ standard configuration ($k_1=1.5, b=0.75, \delta =1.0$). $\text {IDF}_j(t)$ represents inverse document frequency, adopting the smoothed version $\text {IDF}_j(t)=\log \frac{N_j-df_j(t)+0.5}{df_j(t)+0.5}$ to improve numerical stability. This improved BM25F weighted fusion scheme combines practical experience from the information retrieval field, identifying legal professional terms in queries and improving task identification accuracy through term frequency saturation functions and document length normalization mechanisms.

**Algorithm 1:**
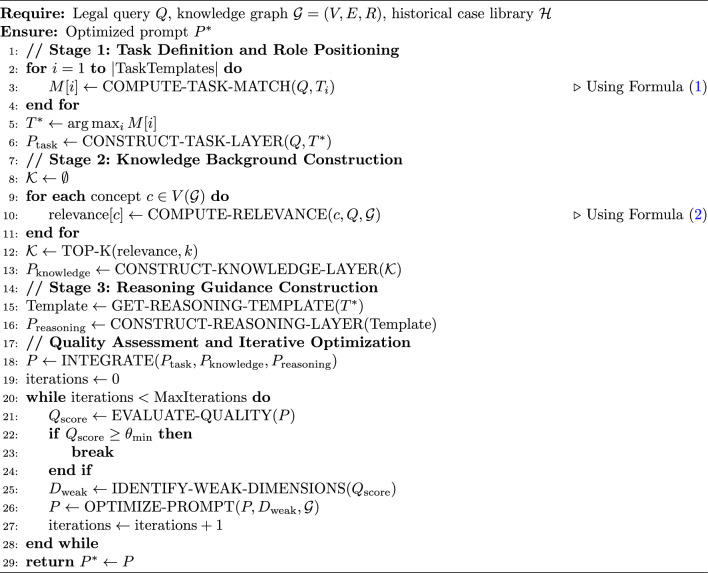
Three-stage hierarchical prompt generation algorithm.

Algorithm 1 presents the three-stage hierarchical prompt generation process. The algorithm first identifies the best task template through a task matching mechanism (lines 2-6), then retrieves relevant legal concepts from the knowledge graph to construct the knowledge background (lines 8-13), next selects an appropriate reasoning template based on task type (lines 15-16), and finally ensures the generated prompt quality reaches preset standards through an iterative optimization loop (up to 3 iterations) (lines 18-26). The algorithm transforms static prompt generation into a dynamic optimization process, capable of adjusting prompt content based on quality assessment feedback, achieving the transformation from “one-time generation” to “continuous improvement.”

#### Legal knowledge background construction mechanism

Legal knowledge background construction calculates the relevance between legal concepts and queries through multi-dimensional assessment, with its core lying in considering four dimensions: text relevance, knowledge graph association, case law weight, and jurisdictional relevance. This multi-dimensional assessment mechanism captures the relevance degree of legal concepts, providing knowledge background for models. The calculation formula is as follows:

2 $$\begin{aligned} \begin{aligned} R(C, Q) =&\alpha \cdot R_{\text {text}}(C, Q) + \beta \cdot R_{\text {kg}}(C, Q) \\&+ \gamma \cdot R_{\text {case}}(C, Q) + \delta \cdot R_{\text {jur}}(C, Q) \end{aligned} \end{aligned}$$

This formula constructs a legal concept relevance assessment system through weighted fusion of relevance scores from four dimensions. Here, $\alpha$, $\beta$, $\gamma$, $\delta$ represent weight coefficients for each dimension, satisfying the weight normalization condition $\alpha + \beta + \gamma + \delta = 1$. Dimension weights are determined through Bayesian optimization methods, using Gaussian processes as surrogate models, with Top-5 hit rate as the optimization objective over 50 iterations on historical query data. The optimized weights in this experiment are: $\alpha =0.30$ (text relevance), $\beta =0.35$ (knowledge graph association), $\gamma =0.25$ (case law weight), $\delta =0.10$ (jurisdictional relevance).

To ensure comparability of scores across dimensions and stability across queries, all subscores are normalized using a global robust normalization strategy before fusion: truncation and linear scaling to the [0, 1] interval based on the 95th and 5th percentiles of training set statistics. This method is more robust than min-max normalization based on single-query candidate sets, avoiding cross-query incomparability and sensitivity to candidate pool size. This multi-dimensional assessment method considers surface text matching and mines structural relationships of legal concepts in knowledge graphs, citation frequencies in judicial practice, and regional applicability scope, thereby ensuring that retrieved legal concepts have semantic relevance and comply with judicial practice requirements.

Text relevance $R_{\text {text}}(C, Q)$ adopts the improved BM25+ algorithm for calculation:

3 $$\begin{aligned} R_{\text {text}}(C,Q)=\text {Norm}\left( \sum _{t\in Q\cap C}\text {IDF}(t)\left[ \frac{f(t,C)(k_1+1)}{f(t,C)+k_1\left( 1-b+b\frac{|C|}{\text {avgdl}}\right) }+\delta \right] \right) \end{aligned}$$

This formula is an enhanced version of the classic BM25 algorithm that improves the low-score bias problem for long documents in traditional BM25 methods by introducing a constant term $\delta \ge 0$. Here, *f*(*t*, *C*) represents the frequency of term *t* in concept *C*, |*C*| represents the document length of concept *C*, $\text {avgdl}$ represents the average concept document length, $k_1 \in [1.2, 2.0]$ represents the term frequency saturation parameter (this study sets $k_1=1.5$), $b \in [0, 1]$ represents the document length normalization parameter (this study sets $b=0.75$), and $\delta$ represents the BM25+ constant term (this study sets $\delta =1.0$). $\text {IDF}(t)=\log \frac{N-df(t)+0.5}{df(t)+0.5}$ represents the smoothed inverse document frequency, where *N* represents the total number of documents and *df*(*t*) represents the number of documents containing term *t*. $\text {Norm}(\cdot )$ represents global robust normalization. The BM25+ algorithm handles legal texts of different lengths through term frequency saturation functions and length normalization mechanisms, avoiding biases that simple term frequency statistics might bring.

Knowledge graph association $R_{\text {kg}}(C, Q)$ is calculated based on path distance between concept nodes:

4 $$\begin{aligned} R_{\text {kg}}(C, Q) = \text {Norm}\left( \text {Top3-Avg}\left( \left\{ \lambda ^{d(C,e)} \cdot \frac{1}{1 + d(C, e)} \,\Big |\, e\in \mathcal {E}_Q\right\} \right) \right) \end{aligned}$$

This formula combines path attenuation factor $\lambda \in (0,1)$ with distance reciprocal, considering both the influence of path length and ensuring that concepts at greater distances are not excluded. Using Top-3 averaging instead of simple maximum operation improves robustness to multi-entity support signals. Here, $\mathcal {E}_Q$ represents the set of legal entities identified from query *Q*, and *d*(*C*, *e*) represents the shortest path length from concept *C* to entity *e* in the knowledge graph. The path attenuation factor is set to $\lambda =2^{-1/4}\approx 0.841$, corresponding to a half-life $H=4$ hops: path weights decay by half approximately every 4 hops. This setting results in weights of approximately 0.841, 0.707, 0.595, 0.500, 0.420 for path lengths of 1, 2, 3, 4, 5 respectively, capturing multi-hop associations while avoiding excessive attenuation. This design captures deep associations between legal concepts—even if two concepts do not directly co-occur at the text level, the system can identify their potential associations as long as there is a path connection between them in the knowledge graph.

Case law weight $R_{\text {case}}(C, Q)$ is calculated based on citation statistics of concepts in relevant precedents, adopting the $\text {Norm}(\log (1+\text {citations}))$ form, suppressing the influence of extreme citation numbers through logarithmic transformation. Jurisdictional relevance $R_{\text {jur}}(C, Q)$ measures the overlap degree of jurisdictional label sets through the Jaccard similarity coefficient. The combination of four dimensions ensures that retrieved legal concepts have semantic relevance and comply with judicial practice requirements, thereby providing legal knowledge background for LLMs.

#### Legal reasoning guidance and professional path templates

The legal reasoning guidance framework adopts a multi-dimensional assessment method to measure the professional level of responses, including five key dimensions: legal accuracy, content comprehensiveness, citation standardization, logical rigor, and professional expression norms. Each dimension is assigned different weights, with quality scores calculated through weighted summation to ensure generated legal analysis meets professional standards. The assessment criteria reference the accuracy dimension in the QUEST evaluation framework, focusing on consistency between AI system outputs and authoritative legal standards.

The quality score calculation formula is as follows:

5 $$\begin{aligned} Q_{\text {score}} = \sum _{i \in \{A,C,S,L,E\}} w_i \cdot \text {Score}_i \end{aligned}$$

This formula assesses the overall quality of legal responses through weighted summation. Dimension weights $w_i$ are determined through the Analytic Hierarchy Process (AHP) and satisfy the weight normalization condition $\sum w_i = 1$. A judgment matrix is constructed for each dimension with pairwise comparison scores by legal experts, with consistency ratio $CR=0.08<0.1$, meeting consistency requirements. The weights determined in this study are: legal accuracy $w_A=0.35$, content comprehensiveness $w_C=0.20$, citation standardization $w_S=0.20$, logical rigor $w_L=0.15$, professional expression $w_E=0.10$.

Dimension scores $\text {Score}_i\in [0,1]$ adopt normalized scales, specifically defined as follows: Legal accuracy (*A*) assesses concept precision scores, calculated as a weighted average of legal terminology accuracy and legal provision citation correctness, with benchmark calibration as relatively accurate (1.0), slight deviations (0.8), partially incorrect (0.5), larger errors (0.2), obviously incorrect (0.0); Content comprehensiveness (*C*) measures the coverage degree of required legal points in responses, calculated as the ratio of actually covered points to total necessary points; Citation standardization (*S*) assesses format correctness and reliability of legal citations, considering citation format standardization (40%), source authoritativeness (40%), and timeliness (20%); Logical rigor (*L*) is assessed through coherence scores between adjacent reasoning steps, using a reasoning chain completeness checking algorithm; Professional expression norms (*E*) assess terminology accuracy, format compliance, and style appropriateness according to legal document writing standards. This five-dimensional assessment system forms a systematic legal response quality evaluation framework for guiding LLMs to generate legal analysis that meets professional standards. In practical applications, quality threshold $\theta _{\min }$ is dynamically set according to task types, with $\theta _{\min }=0.85$ for high-risk legal opinions and $\theta _{\min }=0.75$ for general consultations. Quality threshold settings are based on cost-benefit analysis: through manual review of 200 cases, error rates and iteration costs at different thresholds are statistically analyzed. Results show that high-risk tasks at $\theta _{\min }=0.85$ reduce error rates below 5% with an average of 2.3 iterations; general consultations at $\theta _{\min }=0.75$ balance quality (8% error rate) with efficiency (average 1.6 iterations).

#### Dynamic prompt optimization mechanism

The dynamic prompt optimization mechanism serves as the adaptive component of this framework for addressing the complexity of legal dispute analysis, improving legal analysis quality through continuous monitoring and feedback adjustment. This mechanism adopts a closed-loop design concept, transforming assessment feedback into prompt optimization instructions to achieve system adaptive learning capabilities. During operation, this mechanism exhibits phased characteristics, with initial generation, quality assessment, dynamic adjustment, and regeneration as core steps.

**Fig. 2:**
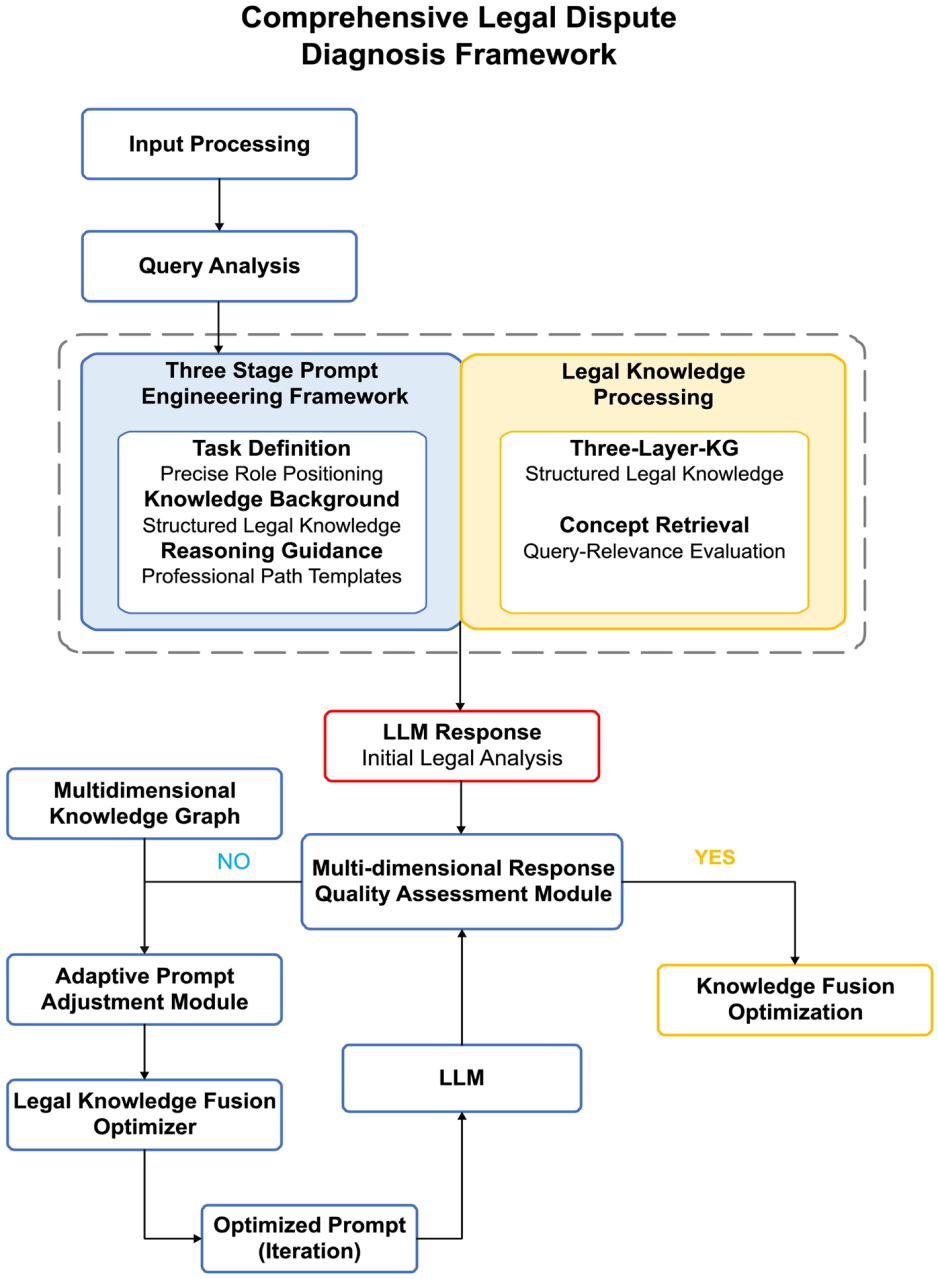
Workflow of the dynamic prompt optimization mechanism.

As shown in Fig. 2, the workflow of the dynamic prompt optimization mechanism begins with user legal query input. The system first generates an initial legal prompt based on query content, guiding LLMs to produce preliminary legal analysis. Subsequently, a multi-dimensional response quality assessment module evaluates the generated content and makes quality judgments based on preset thresholds: if quality meets standards, legal analysis results are output; if quality does not meet standards, an iterative optimization loop is initiated. In the optimization loop, an adaptive prompt adjustment module adjusts prompt structure and content based on quality assessment feedback, while a legal knowledge fusion optimizer provides professional knowledge in combination with multi-dimensional knowledge graphs to jointly form optimized prompts. This entire process constitutes a closed-loop path of “assessment-adjustment-fusion-reassessment,” continuously cycling until generated results meet preset quality standards. This mechanism is suitable for handling complex cases involving multiple intertwined legal concepts, capable of identifying legal reasoning flaws in model responses and improving them through prompt optimization, achieving the evolution from “static prompts” to “dynamic dialogue.”

### Multi-dimensional knowledge graph design and implementation

The complexity of legal domain knowledge representation requires multi-level structural design to cover legal concepts and relationships at different abstraction levels. The multi-dimensional knowledge graph designed in this study includes both traditional knowledge graph node and relationship representations and incorporates multi-granularity concept retrieval mechanisms, capable of dynamically locating relevant legal concepts based on queries. Knowledge-enhanced LLM-based legal knowledge graphs can improve the accuracy and completeness of knowledge representation, providing a knowledge foundation for legal dispute analysis.

#### Three-layer architecture legal knowledge graph

The basic architecture of the legal knowledge graph constructed in this study includes a legal classification ontology layer, legal representation layer, and legal instance layer, achieving coverage from abstract legal concepts to concrete case applications. This three-layer architecture design conforms to the hierarchical organizational characteristics of legal knowledge, differing from traditional flat knowledge graphs by expressing hierarchical relationships between legal concepts. Structured legal prompt frameworks can guide models in systematic legal analysis, demonstrating certain advantages in identifying legal meanings. The importance of multi-level knowledge representation for legal reasoning has been validated in prior research, providing a theoretical foundation for the three-layer architecture design of this study.

The formal definition of the legal knowledge graph is as follows:

6 $$\begin{aligned} \mathcal {G} = (\mathcal {L}_{\text {onto}}, \mathcal {L}_{\text {rep}}, \mathcal {L}_{\text {inst}}, \mathcal {R}) \end{aligned}$$

This formula defines the structure of the legal knowledge graph. Here, $\mathcal {L}_{\text {onto}}$ represents the legal classification ontology layer, defining basic classifications and conceptual relationships of the legal system, including legal department divisions (such as civil law, criminal law, administrative law, etc.), legal relationship types, and basic legal principles, providing a conceptual framework and classification standards for the entire knowledge graph; $\mathcal {L}_{\text {rep}}$ represents the legal representation layer, located in the middle layer, storing specific legal norm content, including legal provisions, judicial interpretations, regulations and rules, etc. Each legal norm node is linked to corresponding ontology concepts and annotated with its effectiveness hierarchy, scope of application, and temporal effectiveness; $\mathcal {L}_{\text {inst}}$ represents the legal instance layer, located at the bottom layer, containing concrete judicial cases, legal consultation instances, and legal application scenarios. Each instance node links to relevant legal norms and ontology concepts, recording the application methods and adjudication results of legal norms in actual scenarios; $\mathcal {R}$ represents the set of inter-layer relationships, $\mathcal {R} = \{r_{\text {is-a}}, r_{\text {part-of}}, r_{\text {regulates}}, r_{\text {cites}},...\}$, defining various semantic relationships between nodes at different levels and within the same level.

#### Multi-granularity legal concept retrieval mechanism

The multi-granularity legal concept retrieval mechanism designed in this study integrates four independent and complementary matching strategies to achieve dynamic retrieval of legal concepts. To control computational complexity, the system adopts a two-stage retrieval strategy: first, rapid fusion of semantic vectors and terminology matching filters out Top-500 candidate concepts, then executes complete four-strategy matching on the candidate set. All strategy scores are normalized to the [0, 1] interval through global robust normalization and merged through adaptive weight fusion to form comprehensive scores, thereby improving retrieval accuracy and professionalism.

**Algorithm 2:**
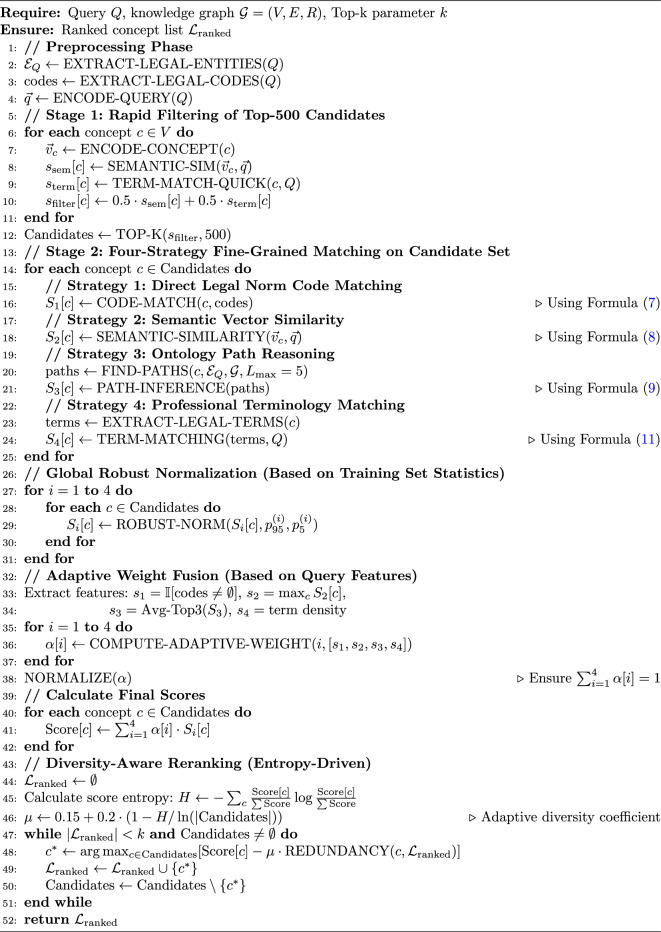
Multi-strategy collaborative legal concept retrieval algorithm.

Algorithm 2 presents the multi-strategy collaborative retrieval process. The algorithm first performs query preprocessing, extracting legal entities, legal codes, and query vectors (lines 2-4). In Stage 1, through a rapid filtering mechanism combining semantic vectors and terminology matching, Top-500 candidates are filtered from concept set *V*, reducing the computational cost of subsequent complex operations (lines 6-13). Stage 2 executes four retrieval strategies in parallel on the candidate set, calculating code matching scores, semantic similarity, path reasoning scores, and terminology matching scores respectively (lines 15-26). All strategy scores are mapped to the [0, 1] interval through global robust normalization (95%/5% percentile truncation) based on training set statistics (lines 28-32), ensuring cross-query comparability. Next, dynamic weights for each strategy are calculated through an adaptive mechanism based on query features (lines 34-39), fusing the four strategy scores to form comprehensive scores (lines 41-43). Finally, reranking is performed through an entropy-driven diversity-aware greedy algorithm, increasing result diversity while ensuring relevance (lines 45-52). This algorithm combines four complementary strategies, controls complexity through two-stage retrieval, adapts to different types of queries through adaptive weight mechanisms, and avoids redundant results through entropy-driven diversity control.

First, the direct matching method based on legal norm codes is calculated as follows:

7 $$\begin{aligned} \text {CM}(C, Q) = \gamma \cdot \mathbb {I}_{\text {exact}}(\text {code}(C), \text {code}(Q)) + (1-\gamma ) \cdot \text {Sim}_{\text {partial}}(\text {code}(C), \text {code}(Q)) \end{aligned}$$

This formula achieves flexible matching of legal codes by combining exact matching and partial matching. Here, $\text {code}(C)$ and $\text {code}(Q)$ represent standardized legal codes (such as legal provision numbers) for concept *C* and query *Q* respectively, $\mathbb {I}_{\text {exact}}$ is an exact match indicator function (1 for match, 0 for no match), $\text {Sim}_{\text {partial}} \in [0,1]$ calculates the degree of partial matching (using longest common subsequence similarity), and $\gamma \in [0,1]$ is a weight coefficient (this study sets $\gamma =0.8$) balancing the importance of exact matching and partial matching. This method is suitable for situations where user queries contain legal provision numbers, enabling location of corresponding legal norms. When queries contain codes such as “Article 577 of the Civil Code,” exact matching returns the corresponding provisions; when queries contain only partial code information, the partial matching mechanism returns relevant sets of legal provisions.

Second, the similarity calculation formula based on legal domain specialized semantic vectors is as follows:

8 $$\begin{aligned} \text {VS}(C, Q) = \frac{\vec {v}_C \cdot \vec {v}_Q}{\Vert \vec {v}_C\Vert \Vert \vec {v}_Q\Vert } \cdot \underbrace{\log \left( 1+\frac{\text {tf}(C)}{\text {df}(C)}\right) }_{\ge 0} \cdot \exp \left( -\frac{d(\text {domain}_C, \text {domain}_Q)}{\sigma }\right) \end{aligned}$$

This formula introduces non-negative Term Frequency-Inverse Document Frequency (TF-IDF) enhancement factors and domain attenuation factors based on traditional cosine similarity. Here, $\vec {v}_C$ and $\vec {v}_Q$ are semantic vector representations generated through legal domain pre-trained models (such as Legal-BERT), with the first term calculating basic cosine similarity; $\text {tf}(C)$ and $\text {df}(C)$ are the term frequency and document frequency of concepts respectively, with the second term ensuring non-negativity through logarithmic transformation $\log (1+\text {tf}/\text {df})$ and boosting weights of important concepts; $d(\cdot , \cdot )$ is a domain distance function (using legal ontology hierarchical distance), $\sigma >0$ is a domain attenuation parameter (this study sets $\sigma =2.0$), and the third term reduces scores of cross-domain concepts through a domain attenuation mechanism. This method captures semantic associations in legal texts, transcending the limitations of surface text matching. Through language models specifically pre-trained on legal corpora, semantic vectors reflect relationships between legal concepts, including hypernym-hyponym relationships, synonym relationships, and association relationships.

Third, the path reasoning score calculation formula based on legal ontology relationship graphs is as follows:

9 $$\begin{aligned} \text {PI}(C, Q) = \max _{e \in \mathcal {E}_Q} \max _{p \in \mathcal {P}(C,e)} \left[ \lambda ^{|p|} \cdot \sum _{r \in p} w_r \cdot \text {Coherence}(p) \right] \end{aligned}$$

This formula calculates concept relevance through valid paths in the knowledge graph, capable of discovering deep associations that are not easily perceptible at the text level. Here, $\mathcal {E}_Q = \{e_1, e_2,..., e_m\}$ is the set of legal entities identified from query *Q*, $\mathcal {P}(C,e)$ is the set of paths from concept *C* to entity *e* (path length $|p|\le L_{\max }$, this study sets $L_{\max }=5$), $\lambda \in (0,1)$ is a path length attenuation factor (this study sets $\lambda =0.841$, corresponding to half-life $H=4$ hops), controlling the influence of path length on scores. *p* is a path from *C* to *e*, $w_r$ is the weight of relationship *r* on the path, reflecting the importance of different relationship types (e.g., is-a relationship weight 0.9, cites relationship weight 0.7). This method utilizes structural information of knowledge graphs to infer potential legal associations through path relationships between concepts, suitable for handling complex queries requiring cross-domain legal knowledge associations.

The path coherence function is defined as:

10 $$\begin{aligned} \text {Coherence}(p) = \left( \prod _{i=1}^{|p|-1} \tilde{c}_{i,i+1}\right) ^{\frac{1}{|p|-1}}, \quad \tilde{c}_{i,i+1}=\frac{\exp (\text {Compat}(r_i, r_{i+1}))}{\sum _{r' \in \mathcal {R}} \exp (\text {Compat}(r_i, r'))} \end{aligned}$$

This formula evaluates the logical coherence of entire paths by calculating the compatibility of adjacent relationship types in paths, adopting geometric mean form to prevent numerical underflow. Here, $\text {Compat}(r_i, r_{i+1})$ represents elements of the relationship compatibility matrix, measuring logical consistency between continuous relationship types. The compatibility matrix is constructed based on legal expert knowledge, reflecting the reasonableness degree of different relationship type combinations. For example, an “is-a” relationship followed by a “part-of” relationship has high compatibility (0.8), while a “cites” relationship followed by an “is-a” relationship has low compatibility (0.2). $\tilde{c}_{i,i+1}$ ensures coherence scores are in the (0, 1] range through softmax normalization. The geometric mean ensures $\text {Coherence}(p)\in (0,1]$ without systematic attenuation as path length increases. This design ensures the system considers both path existence and evaluates path logical reasonableness, thereby improving the reliability of reasoning results.

Fourth, the score calculation formula based on professional terminology matching is as follows:

11 $$\begin{aligned} \text {TM}(C, Q) = \frac{\sum _{t \in \text {Terms}(C)} w_t \cdot \text {Match}(t, Q) \cdot \text {ILT}(t) \cdot \text {Context}(t, C)}{\sum _{t \in \text {Terms}(C)} w_t} \end{aligned}$$

This formula achieves matching of legal professional terminology by considering term matching degree, legal professional weight, and contextual relevance. Here, $\text {Terms}(C)$ is the set of professional legal terms extracted from concept *C*, and $w_t$ is the importance weight of term *t* (calculated through TF-IDF). Term matching degree $\text {Match}(t, Q)$ is defined as:

12 $$\begin{aligned} \text {Match}(t, Q) = \alpha _1 \cdot \mathbb {I}_{\text {exact}}(t, Q) + \alpha _2 \cdot \text {Sim}_{\text {stem}}(t, Q) + \alpha _3 \cdot \text {Sim}_{\text {sem}}(t, Q) \end{aligned}$$

This formula combines exact matching, stemming matching, and semantic matching. Here, $\mathbb {I}_{\text {exact}}(t, Q)\in \{0,1\}$ is the exact match score, $\text {Sim}_{\text {stem}}(t, Q)\in [0,1]$ is the stemming match score (using the Porter stemming algorithm), $\text {Sim}_{\text {sem}}(t, Q)\in [0,1]$ is the semantic similarity score (based on word vector cosine similarity), and $\alpha _1, \alpha _2, \alpha _3$ are weight coefficients (this study sets $\alpha _1=0.6, \alpha _2=0.2, \alpha _3=0.2$), satisfying $\alpha _1 + \alpha _2 + \alpha _3 = 1$. The combination of three matching methods can handle different forms of terminology expression, capable of identifying identical terms and handling word form variations and synonymous expressions.

The term legal professional weight $\text {ILT}(t)$ (Inverse Legal Terminology) is calculated as follows:

13 $$\begin{aligned} \text {ILT}(t) = \log \left( \frac{\text {freq}_{\text {legal}}(t) + \sigma }{\text {freq}_{\text {general}}(t) + \sigma }\right) \cdot \text {JurScope}(t) \end{aligned}$$

This formula identifies terms with legal professional characteristics by comparing the frequency distribution of terms in legal corpora and general corpora. Here, $\text {freq}_{\text {legal}}(t)$ is the frequency of term *t* in legal corpora, $\text {freq}_{\text {general}}(t)$ is the frequency in general corpora, $\sigma$ is a smoothing factor (this study sets $\sigma =1.0$) to avoid computational problems caused by zero frequency. $\text {JurScope}(t) \in [0,1]$ is the term’s judicial application scope coefficient, defined as $\text {JurScope}(t)=1-\frac{\text {Var}(\text {usage}_{\text {jurisdictions}}(t))}{\max _{\text {var}}}$, where $\text {Var}(\cdot )$ is variance and $\max _{\text {var}}$ is a normalization constant, reflecting the consistency of term application across different judicial domains. This method identifies typical legal terms such as “bona fide acquisition” and “gross negligence” and assigns higher weights, thereby improving retrieval professionalism.

The context score function is defined as:

14 $$\begin{aligned} \text {Context}(t, C) = \frac{1}{|\text {Window}(t,C)|} \sum _{w \in \text {Window}(t,C)} \text {PMI}(t, w) \cdot \mathbb {I}[w \in \mathcal {V}_{\text {legal}}] \end{aligned}$$

This formula captures the contextual semantic features of terms through Pointwise Mutual Information (PMI). Here, $\text {Window}(t,C)$ is the context window of term *t* in concept *C* (5 words before and after), $\text {PMI}(t, w) = \log \frac{p(t,w)}{p(t) \cdot p(w)}$ is pointwise mutual information measuring co-occurrence strength between two words, $\mathcal {V}_{\text {legal}}$ is a legal professional vocabulary (containing approximately 10,000 commonly used legal terms), and $\mathbb {I}[\cdot ]\in \{0,1\}$ is an indicator function. This method distinguishes the professional degree of the same term in different contexts. For example, “good faith” in the “bona fide acquisition” context has higher PMI values than in the “kind reminder” context, thereby ensuring the system identifies legal professional usage.

The diversity-aware redundancy calculation formula is:

15 $$\begin{aligned} \text {Redundancy}(c, \mathcal {L}) = \max _{c' \in \mathcal {L}} \left[ \omega _1 \cdot \text {Sim}_{\text {sem}}(c,c') + \omega _2 \cdot \text {Overlap}_{\text {dom}}(c,c') + \omega _3 \cdot \text {Sim}_{\text {struct}}(c,c')\right] \end{aligned}$$

This formula judges the degree of redundancy between concepts by synthesizing similarities in semantic, domain, and structural dimensions. Here, $\omega _1, \omega _2, \omega _3$ are dimension weights (this study sets $\omega _1=0.4, \omega _2=0.3, \omega _3=0.3$), and $\text {Sim}_{\text {sem}}\in [0,1]$ is semantic similarity (using the cosine part of Formula (8)). $\text {Overlap}_{\text {dom}}(c,c')\in [0,1]$ is domain overlap degree, defined as:

16 $$\begin{aligned} \text {Overlap}_{\text {dom}}(c,c') = \frac{|\text {Domain}(c) \cap \text {Domain}(c')|}{|\text {Domain}(c) \cup \text {Domain}(c')|} \end{aligned}$$

Here, $\text {Domain}(c)$ is the set of legal domain labels to which concept *c* belongs (such as {civil law, contract law, tort law}), calculating set overlap degree using the Jaccard coefficient. $\text {Sim}_{\text {struct}}\in [0,1]$ is structural similarity, calculated using the Jaccard coefficient:

17 $$\begin{aligned} \text {Sim}_{\text {struct}}(c_1,c_2) = \frac{|\mathcal {N}(c_1) \cap \mathcal {N}(c_2)|}{|\mathcal {N}(c_1) \cup \mathcal {N}(c_2)|} \end{aligned}$$

Here, $\mathcal {N}(c)$ represents the set of neighbor nodes of concept *c* in the knowledge graph (1-hop neighbors). This method measures the similarity degree of two concepts in graph structure by calculating the proportion of common neighbors to all neighbors. The diversity control mechanism avoids returning similar concepts such as “tort liability” and “tortious conduct,” instead ensuring the return of concepts representing different legal relationships such as “breach of contract” and “tort liability,” thereby providing users with a more comprehensive legal knowledge perspective.

This multi-strategy fusion mechanism utilizes the structured features and semantic associations of legal texts, enabling identification of legal concepts relevant to queries and providing a knowledge foundation for legal dispute analysis. The system ranks retrieved legal concepts based on comprehensive scores, prioritizing the presentation of more relevant content, thereby improving the accuracy and professionalism of legal analysis.

#### Integration of knowledge graph and web search

The timeliness issue of legal knowledge is a key technical challenge for legal LLMs, with traditional static knowledge representation struggling to adapt to frequent updates of legal regulations and dynamic evolution of judicial interpretations. This framework constructs a multi-source heterogeneous legal knowledge integration mechanism, achieving coupling of multi-dimensional knowledge graphs with professional legal web search. Legal knowledge graphs as an intermediate layer between users and LLMs can enhance the legal correctness and citation standardization of model answers.

This mechanism first establishes a unified retrieval interface covering authoritative legal data sources, ensuring that retrieved legal information meets timeliness requirements through three mechanisms: the jurisdictional identification mechanism judges the applicable legal system and regional scope based on query content; the legal concept timeliness marking mechanism annotates each legal concept in the knowledge graph with effective time, ineffective time, and revision history; the change tracking mechanism monitors revisions and abolitions of legal regulations in real-time, updating the knowledge graph in a timely manner. Second, this study develops a legal authoritativeness assessment model that calculates authoritativeness scores based on factors such as legal source types, publishing institution hierarchy, and citation frequency, prioritizing the use of high-authority information. Finally, semantic fusion of knowledge graphs with dynamic retrieval results is achieved, forming a legal knowledge service system that maintains structured representation advantages while having real-time update capabilities.

#### Core parameter configuration and reproducibility

To ensure system reproducibility, Table 1 summarizes core parameter configurations for each module, including default values and experimentally optimized values. These parameters have undergone systematic experimental validation and expert review, balancing retrieval accuracy while considering computational efficiency.

**Table 1:** Overview of core parameter configurations.

Module	Parameter	Optimized Value/Range
Task Matching	Text field	$k_1=1.5, b=0.75, \delta =1.0$
	Code field	$k_1=1.2, b=0.0, \delta =0.3$
	Dimension weights	(0.35, 0.28, 0.22, 0.15)
Knowledge Background	Fusion weights	$(\alpha ,\beta ,\gamma ,\delta )=(0.30, 0.35, 0.25, 0.10)$
	BM25+	$k_1=1.5, b=0.75, \delta =1.0$
Graph Retrieval	Path attenuation	$\lambda =0.841$ (half-life $H=4$, range 3-5)
	Domain attenuation	$\sigma =2.0$ (range 1.5-3.0)
	Candidate pool	Top-500 (range 200-1000)
Term Matching	Match weights	$(\alpha _1,\alpha _2,\alpha _3)=(0.6, 0.2, 0.2)$
	ILT smoothing	$\sigma =1.0$
	Context window	5 words before/after
Diversity Reranking	Base coefficient	$\mu \in [0.15, 0.35]$ (adaptive)
	Top-k	$k=12$
Quality Assessment	Dimension weights	(0.35, 0.20, 0.20, 0.15, 0.10)
	Quality threshold	$\theta _{\min }=0.85$(high-risk), 0.75(general)

Sensitivity analysis of path attenuation factor $\lambda$ and domain attenuation parameter $\sigma$ shows performance differences within the recommended range are less than 2%, demonstrating parameter selection robustness. Quality threshold settings are based on cost-benefit analysis: high-risk tasks at $\theta _{\min }=0.85$ reduce error rates below 5%, while general consultations at $\theta _{\min }=0.75$ balance quality with efficiency.

## Experiments and evaluation

### Statistical analysis methods

To ensure the scientific rigor and reliability of experimental results, we adopt a strict statistical analysis framework. Paired t-tests are used to assess performance differences between baseline and complete configurations, with significance level set at $\alpha =0.05$. For non-normally distributed data, the Wilcoxon signed-rank test is employed. Each model configuration runs 3 times on 500 test samples (random seeds: 42, 2024, 2025), reporting means and 95% confidence intervals. Effect size is measured using Cohen’s d, with judgment criteria: small effect ($d=0.2$), medium effect ($d=0.5$), large effect ($d=0.8$), very large effect ($d>2.0$). In ablation experiments, Bonferroni correction controls family-wise error rate in multiple comparisons. All experiments are conducted on two NVIDIA A100 (80GB) Graphics Processing Units (GPUs), with statistical analysis completed using Python scipy.stats package.

### Experimental setup

**Dataset construction and sampling strategy:** To ensure comprehensive evaluation and cross-jurisdictional adaptability of the framework, this study conducts integrated sampling from six internationally recognized legal AI benchmark datasets, including COLIEE 2024^36^ (Canadian Supreme Court cases), LegalBench^2^ (common law system comprehensive benchmark), LeCaRDv2^37^ (Chinese criminal cases), LexGLUE^38^ (European and American multi-national legal texts), ECHR^39^ (European Court of Human Rights cases), and JEC-QA^40^ (Chinese National Judicial Examination Q&A). The sampling process follows a strict three-dimensional stratified random sampling method: first, sample allocation by jurisdictional balance, with common law systems (United States, Canada, United Kingdom) accounting for 35%, civil law systems (China, European Union) accounting for 45%, and mixed/international law systems accounting for 20%, reflecting the actual distribution of global legal systems; second, ensuring legal domain diversity, covering 13 major classifications including intellectual property, contract law, tort law, labor law, property law, corporate law, administrative law, civil law, international law, criminal law, family law, constitutional law, and tax law, with each domain containing 30-50 samples; finally, ensuring task type completeness, including four core tasks: case retrieval (30%), judgment prediction (25%), legal text entailment (25%), and legal Q&A reasoning (20%). Samples are categorized by complexity into basic level (30%, single legal concepts), intermediate level (50%, requiring 2-3 step reasoning), and advanced level (20%, complex legal disputes requiring 4+ step reasoning), with complexity independently assessed by two law professors who resolve disagreements through discussion. Ultimately, 500 pairs of high-quality Q&A pairs are extracted from the above datasets to constitute the test set, ensuring balanced domain distribution and diverse case complexity.

**Annotation Protocol and Quality Control:** All reference answers and manual assessments are completed by legal professionals with qualifications. The annotation team includes 5 practicing lawyers (average 8.2 years of practice), 3 law professors, and 2 senior judges (12-15 years of adjudication experience). The annotation process adopts a strict three-stage workflow: first, each sample is independently annotated by at least 3 experts to avoid anchoring effects; then, Inter-Annotator Agreement (IAA) is calculated, evaluated using Fleiss’ Kappa coefficient and Intraclass Correlation Coefficient (ICC); finally, expert meetings are organized to reach consensus on cases with consistency below the threshold (Fleiss’ Kappa < 0.6). The annotation manual provides detailed standards for five evaluation dimensions (legal accuracy, content comprehensiveness, citation standardization, logical rigor, professional expression), as well as boundary case handling procedures and unified citation format specifications. IAA analysis based on 30 calibration samples shows Fleiss’ Kappa coefficients for the five dimensions range from 0.58-0.81 (overall average 0.69), reaching “substantial agreement” level^41^; ICC(2,10) averages 0.96, indicating good reliability of collective ratings by 10 raters; Cronbach’s $\alpha$ coefficient averages 0.91, confirming internal consistency of the assessment tool. The legal accuracy and citation standardization dimensions exhibit higher consistency ($\kappa$=0.74 and 0.81), reflecting convergence of legal professionals’ judgments on objective legal standards; the logical rigor dimension shows relatively lower consistency ($\kappa$=0.58), mainly stemming from subjective judgment differences among experts regarding reasoning path merits. Twelve percent of cases triggered collective discussion mechanisms due to initial rating differences exceeding 20 points (100-point scale) and reached consensus. These difficult cases were mainly concentrated in labor law and property law domains, reflecting the contextualized nature of knowledge in these fields. Quality assurance mechanisms also include blind testing design (raters unaware of sample sources and other rating results), regular calibration meetings (unified standards every 100 samples annotated), and difficult case library construction (recording low-consistency cases and consensus results as training materials), ensuring dataset quality and reliability.

To evaluate framework universality, this study selects four representative LLMs for experiments: DeepSeek-R1-70B^42^ (70B parameters), Qwen3-Next-80B^43^ (80B parameters), Llama 4 Scout-109B^44^ (109B parameters), and gpt-oss-120b (120B parameters). Experiments adopt a frozen-parameter zero-shot prompting paradigm, with all LLM pre-training parameters remaining unchanged, without any form of fine-tuning, parameter-efficient fine-tuning (such as LoRA, Prefix-tuning), or continual learning. All experiments are conducted in identical computational environments using two NVIDIA A100 (80GB) GPUs, ensuring result fairness and comparability. Each model configuration runs 3 times on 500 test samples (random seeds: 42, 2024, 2025), reporting means and 95% confidence intervals. Performance analysis shows baseline configuration average inference time is 2.3 seconds/query, complete configuration is 3.8 seconds/query, with additional overhead mainly from knowledge graph retrieval and prompt construction processes.

### Main results

#### BLEU and ROUGE metric evaluation

We first assess framework performance in text generation quality using the widely adopted BLEU and ROUGE metric families. Experiments are conducted on four representative models, with each model running 3 times to ensure result stability. Table 2 presents baseline versus complete configuration performance comparisons across multiple n-gram granularities and recall dimensions, revealing systematic improvements in text generation capabilities by the framework.

**Table 2:** Model performance comparison on BLEU and ROUGE metrics.

Model	BLEU-1		BLEU-2		BLEU-3		BLEU-4	
	Baseline	Complete	Baseline	Complete	Baseline	Complete	Baseline	Complete
DeepSeek-R1-70B	0.2806	0.5428	0.2154	0.4836	0.1749	0.4322	0.1461	0.3968
Qwen3-Next-80B	0.2632	0.6535	0.2050	0.5969	0.1669	0.5474	0.1395	0.5075
Llama 4 Scout-109B	0.2445	0.6247	0.1916	0.5619	0.1549	0.4887	0.1282	0.4427
gpt-oss-120b	0.2529	0.6070	0.1925	0.5468	0.1517	0.4959	0.1239	0.4580

	ROUGE-1 F1		ROUGE-2 F1		ROUGE-L F1			
	Baseline	Complete	Baseline	Complete	Baseline	Complete		
DeepSeek-R1-70B	0.3823	0.6609	0.2497	0.5476	0.3453	0.6566		
Qwen3-Next-80B	0.3790	0.7453	0.2481	0.6433	0.3438	0.7425		
Llama 4 Scout-109B	0.3599	0.7442	0.2336	0.6302	0.3238	0.7437		
gpt-oss-120b	0.3642	0.7059	0.2277	0.6060	0.3249	0.7031		

**Fig. 3:**
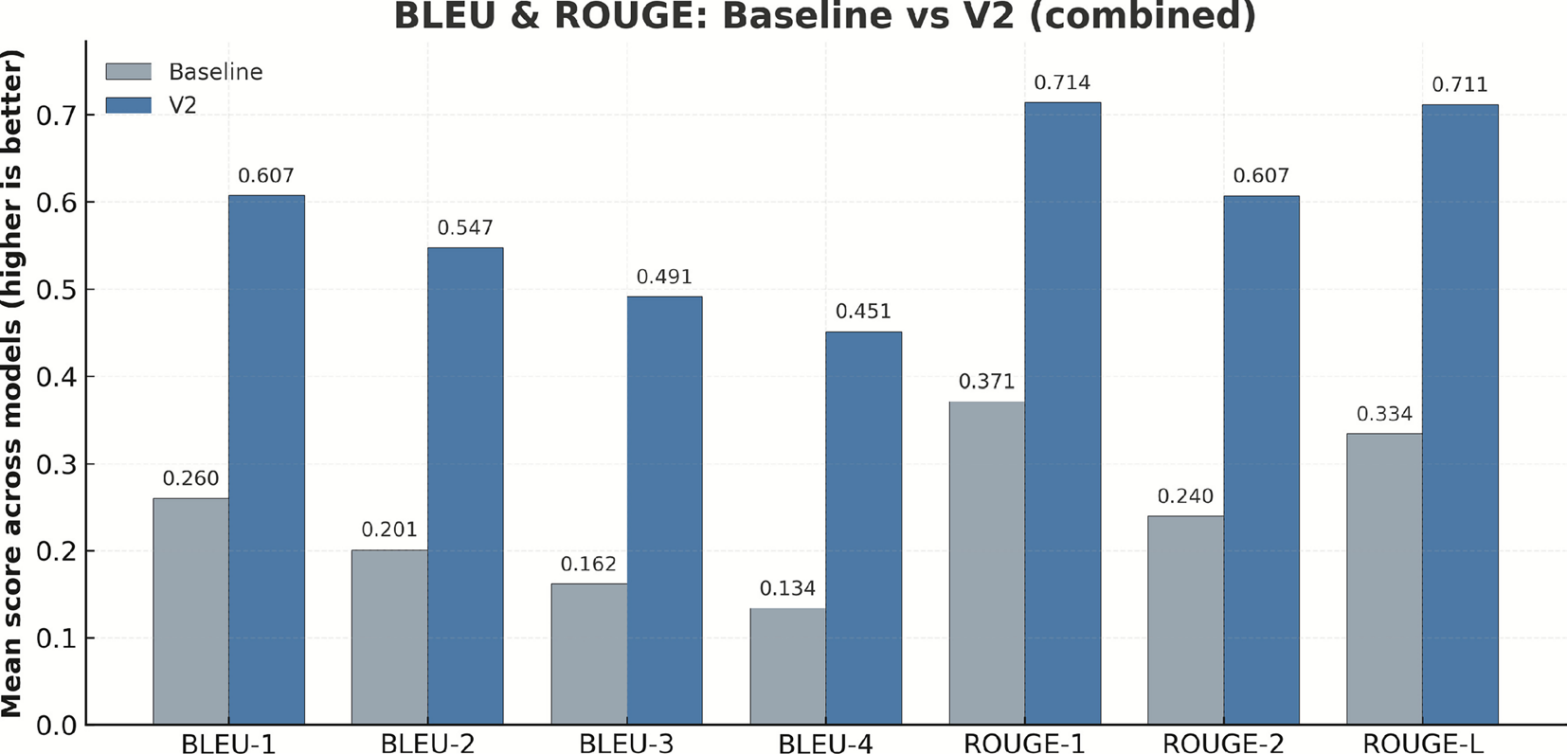
Comprehensive comparison of BLEU and ROUGE metrics: average performance of baseline versus complete configurations.

As shown in Fig. 3, the complete configuration (deep blue) surpasses the baseline configuration (light gray) across all BLEU and ROUGE metrics. The BLEU-4 metric increased from 0.134 to 0.451 (gain of 0.317). This incremental improvement pattern from low-order to high-order n-gram metrics indicates that the framework improves lexical selection accuracy and enhances the model’s ability to construct complex syntactic structures. Synchronous ROUGE metric improvements confirm framework effectiveness: ROUGE-1, ROUGE-2, and ROUGE-L reached 0.714, 0.607, and 0.711 respectively, with ROUGE-L F1 improving from 0.334 to 0.711 (gain of 0.377). This multi-dimensional coordinated improvement indicates that generated texts maintain quality at local vocabulary and phrase levels and achieve improvements in global structure and long-distance dependency relationships.

#### Key legal performance metric evaluation

We assess framework performance in core legal judgment capabilities using four key metrics: F1 score, Exact Match (EM), Macro F1, and Micro F1. These metrics measure model capabilities in legal concept identification, answer accuracy, and category balance from different perspectives. Table 3 presents statistical evaluation results on 500 test samples, with each configuration running 3 times reporting mean ± standard deviation.

**Table 3:** Statistical evaluation results of legal LLMs on key performance metrics$$^{***}$$.

Metric	Baseline	Complete	Absolute Gain	Relative Gain	95% CI	Cohen’s *d*
**F1 Score**	0.356±0.011	**0.714±0.043**	0.358	+0.358	[0.312, 0.406]	7.50
**Exact Match**	0.011±0.010	**0.446±0.054**	0.435	+0.435	[0.381, 0.489]	8.95
**Macro F1**	0.010±0.004	**0.305±0.030**	0.295	+0.295	[0.265, 0.325]	9.83
**Micro F1**	0.011±0.005	**0.445±0.055**	0.434	+0.434	[0.379, 0.489]	8.71

$$^{***}$$All metrics reached $$p<0.001$$ significance level (paired t-test, $$n=500$$, 3 runs).

Cohen’s $d>$7.5 indicates large effect size, 95% CI shows stable improvement.

Cohen’s d calculated using pooled standard deviation of paired samples: $d = \frac{M_{Complete} - M_{Baseline}}{SD_{pooled}}$.

Results presented in Table 3 reveal framework progress in core legal judgment capabilities. The F1 score improvement (from 0.356 to 0.714) combined with narrow confidence interval [0.312, 0.406] confirms improvement stability and reliability. The exact match metric improvement from near-zero baseline (0.011) to 0.446 (gain of 0.435) means the complete configuration can generate accurate legal answers for a substantial portion of queries. Synchronous improvements in Macro F1 and Micro F1 (gains of 0.295 and 0.434 respectively) indicate framework robustness in handling imbalanced legal categories, improving both common case type performance and rare legal problem handling capabilities. Cohen’s d effect sizes all exceed 7.5, far greater than the traditional “large effect” (0.8) standard, confirming practical significance of improvements.

#### Calibration error analysis

Model calibration quality reflects consistency between prediction confidence and actual accuracy, crucial for legal AI system trustworthiness. We adopt Expected Calibration Error (ECE) and Maximum Calibration Error (MCE) metrics to assess model self-awareness degree. Table 4 presents calibration performance comparison of four models under baseline and complete configurations.

**Table 4:** Model calibration error comparison.

Model	ECE		MCE	
	Baseline	Complete	Baseline	Complete
DeepSeek-R1-70B	0.2351	0.1978	0.2356	0.2956
Qwen3-Next-80B	0.2755	0.2785	0.2758	0.5766
Llama 4 Scout-109B	0.3052	0.1756	0.3053	0.1758
gpt-oss-120b	0.3107	0.3749	0.3166	0.9546

Llama 4 Scout-109B exhibits better calibration performance under complete configuration, with expected calibration error decreasing from 0.3052 to 0.1756 (reduction of 0.1296). This improvement mainly benefits from structured knowledge support provided by the framework—when models can access legal concept definitions and relationships, their assessment of prediction reliability also becomes more reasonable. In contrast, gpt-oss-120b’s maximum calibration error increased from 0.3166 to 0.9546. In-depth analysis reveals this phenomenon mainly occurs when handling rare legal categories; the model becomes overconfident about certain edge cases after obtaining knowledge enhancement, suggesting we need to set differentiated confidence calibration strategies for different model architectures when deploying legal AI systems.

#### Domain-specific performance analysis

To evaluate framework adaptation capabilities across different legal domains, we conduct tests on 13 major legal classifications. Each domain contains 30-50 carefully selected cases, covering from structured European Union (EU) law to contextually rich labor law. Table 5 presents baseline versus complete configuration EM and F1 score comparisons across domains, revealing framework domain-specific performance characteristics. The performance shown here is for a single best model (Qwen3-Next-80B) across domains to more clearly reflect inter-domain differences.

**Table 5:** Performance comparison across different legal domains (Qwen3-Next-80B model)$^{\dagger }$.

Legal Domain	EM		F1	
	Baseline	Complete	Baseline	Complete
EU Law	0.373	0.714	0.416	0.886
Intellectual Property	0.410	0.844	0.582	0.829
General Legal	0.265	0.455	0.396	0.768
Corporate Law	0.212	0.625	0.305	0.757
Administrative Law	0.121	0.563	0.309	0.748
Civil Law	0.214	0.586	0.325	0.729
International Law	0.132	0.355	0.333	0.701
Criminal Law	0.183	0.424	0.305	0.675
Family Law	0.282	0.509	0.276	0.663
Constitutional Law	0.026	0.359	0.354	0.659
Tax Law	0.123	0.443	0.419	0.598
Employment Law	0.125	0.125	0.433	0.571
Property Law	0.044	0.167	0.303	0.523

$^{\dagger }$This table shows single best model performance across domains. Table 3 shows four-model averages, where baseline EM is lower mainly because the other three models approach zero in most domains.

**Fig. 4:**
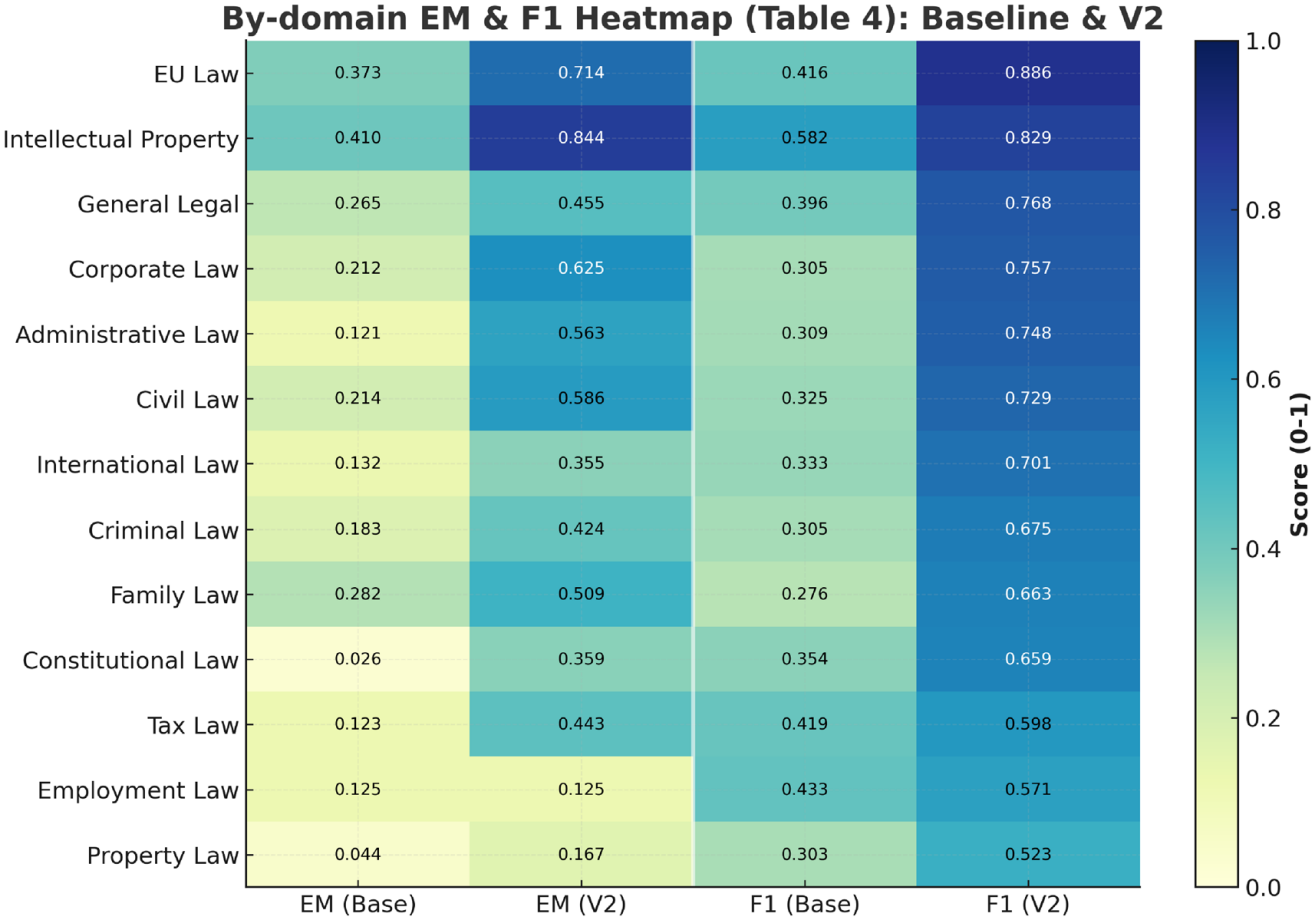
EM and F1 score heatmap across legal domains: baseline versus complete configuration comparison.

As shown in Fig. 4, the heatmap displays framework performance improvement patterns across 13 legal domains through color depth, with gradients from light yellow (low performance) to deep blue (high performance) reflecting complete configuration improvement effects across all domains. Vertical comparison of the heatmap (Baseline EM vs Complete EM, Baseline F1 vs Complete F1) shows a consistent color deepening trend, confirming framework universal effectiveness. EU law and intellectual property law present deeper blue under complete configuration (Complete EM: 0.714 and 0.844 respectively, Complete F1: 0.886 and 0.829 respectively), which relates to these two domains’ knowledge representation characteristics: EU law benefits from its unified legal framework, with this structure fitting with this study’s three-layer knowledge graph architecture; intellectual property law’s success stems from its relatively independent conceptual system. In contrast, labor law and property law show improvement but colors remain relatively lighter, with labor law EM maintaining 0.125 unchanged, reflecting this domain’s contextualized knowledge characteristics, involving specific employment relationships, industry practices, and local regulations difficult to fully capture through static knowledge graphs. Despite these domain differences, the framework achieves positive improvements across all 13 domains, with average F1 score improving from 0.366 to 0.700 (gain of 0.334), validating broad applicability of the multi-dimensional knowledge graph and prompt engineering combination strategy.

#### Cross-jurisdictional performance analysis

Jurisdictional differences in legal systems are key challenges for global legal AI applications. We test framework adaptation capabilities across 8 different types of jurisdictions, including common law systems, civil law systems, mixed law systems, and religious law systems. Table 6 presents citation pattern recognition accuracy and model confidence across different jurisdictional types, revealing framework understanding capabilities of legal source diversity.

**Table 6:** Citation pattern and confidence analysis across jurisdictional types.

Jurisdictional System Type	Primary Citation Type	Average Confidence
Common Law (US Federal)	Case	0.945
Common Law (UK)	Statute	0.917
Civil Law (Germany)	Constitution	0.914
Civil Law (EU)	Case	0.933
Mixed Law (India)	Constitution	0.950
Mixed Law (South Africa)	Statute	0.965
Local Regulations (US State)	Regulation	0.892
Religious Law	Doctrine	0.927

Citation type distribution reflects characteristics of each legal system: the United States and EU primarily rely on case law, embodying the core position of the precedent binding principle in case law systems; the UK and South Africa emphasize statutes, reflecting recent statutory reform trends in these jurisdictions; Germany and India more frequently cite constitutional provisions, embodying the importance continental law countries place on fundamental law. South Africa achieves high confidence of 0.965, possibly related to that country’s mixed legal system characteristics—combining Roman-Dutch law, English common law, and indigenous customary law provides rich association paths for knowledge graphs. Overall average confidence of 0.924 confirms framework cross-jurisdictional adaptability, valuable for handling cross-border legal disputes and comparative law research.

#### Ablation experiment analysis

To systematically verify relative contributions of framework technical components, we design five ablation configurations, each removing or replacing one key component. Experiments are conducted on four representative models, with each configuration running 3 times taking averages. Table 7 presents detailed ablation experiment results, quantifying each component’s impact on overall performance.

**Table 7:** Detailed ablation experiment results (complete data for four models).

Model	Configuration	EM	F1	Macro F1	Micro F1	BLEU-1	BLEU-2	BLEU-3	BLEU-4	ROUGE-1	ROUGE-2	ROUGE-L
*DeepSeek-R1-70B*												
	Baseline	0.0019	0.3650	0.0056	0.0115	0.2806	0.2154	0.1749	0.1461	0.3823	0.2497	0.3453
	Ablation 1: No reasoning guidance	0.3457	0.6278	0.2565	0.3456	0.5047	0.4526	0.3994	0.3669	0.6299	0.5202	0.6258
	Ablation 2: Static weights	0.3802	0.6294	0.2754	0.3834	0.5163	0.4644	0.4187	0.3872	0.6323	0.5307	0.6288
	Ablation 3: No path reasoning	0.2743	0.5765	0.2126	0.2712	0.4662	0.4194	0.3790	0.3549	0.5805	0.4878	0.5769
	Ablation 4: No iterative optimization	0.3552	0.6255	0.2620	0.3552	0.5098	0.4551	0.4140	0.3882	0.6285	0.5257	0.6247
	Ablation 5: Flattened structure	0.3676	0.6268	0.2647	0.3632	0.5191	0.4732	0.4316	0.4038	0.6301	0.5420	0.6255
	**Complete configuration**	**0.3783**	**0.6586**	**0.2701**	**0.3775**	**0.5428**	**0.4836**	**0.4322**	**0.3968**	**0.6609**	**0.5476**	**0.6566**
*Qwen3-Next-80B*												
	Baseline	0.0053	0.3667	0.0126	0.0066	0.2632	0.2050	0.1669	0.1395	0.3796	0.2481	0.3438
	Ablation 1: No reasoning guidance	0.3953	0.6982	0.2832	0.3952	0.5445	0.4935	0.4501	0.4159	0.6970	0.5860	0.6945
	Ablation 2: Static weights	0.4307	0.7292	0.3007	0.4385	0.6275	0.5607	0.5088	0.4673	0.7328	0.6164	0.7305
	Ablation 3: No path reasoning	0.4924	0.7517	0.3289	0.4935	0.6586	0.5849	0.5245	0.4799	0.7513	0.6302	0.7497
	Ablation 4: No iterative optimization	0.4367	0.7202	0.3007	0.4364	0.6203	0.5597	0.5107	0.4708	0.7239	0.6162	0.7222
	Ablation 5: Flattened structure	0.4354	0.7316	0.3031	0.4357	0.6275	0.5670	0.5168	0.4752	0.7336	0.6230	0.7313
	**Complete configuration**	**0.5051**	**0.7479**	**0.3355**	**0.5056**	**0.6535**	**0.5969**	**0.5474**	**0.5075**	**0.7453**	**0.6433**	**0.7425**
*Llama 4 Scout-109B*												
	Baseline	0.0174	0.3449	0.0093	0.0150	0.2445	0.1916	0.1549	0.1282	0.3599	0.2336	0.3238
	Ablation 1: No reasoning guidance	0.3855	0.6524	0.2786	0.3850	0.4948	0.4280	0.3647	0.3279	0.6496	0.5199	0.6475
	Ablation 2: Static weights	0.4079	0.6987	0.2857	0.4453	0.5787	0.5131	0.4522	0.4078	0.6997	0.5794	0.6983
	Ablation 3: No path reasoning	0.4358	0.7211	0.3031	0.4353	0.6187	0.5531	0.4899	0.4488	0.7214	0.6017	0.7194
	Ablation 4: No iterative optimization	0.4899	0.6896	0.2857	0.4865	0.5641	0.5014	0.4335	0.3889	0.6899	0.5711	0.6893
	Ablation 5: Flattened structure	0.4256	0.7023	0.2958	0.4254	0.5839	0.5203	0.4523	0.4075	0.7041	0.5863	0.7028
	**Complete configuration**	**0.4557**	**0.7475**	**0.3127**	**0.4553**	**0.6247**	**0.5619**	**0.4887**	**0.4427**	**0.7442**	**0.6302**	**0.7437**
*gpt-oss-120b*												
	Baseline	0.0232	0.3466	0.0134	0.0185	0.2529	0.1925	0.1517	0.1239	0.3642	0.2277	0.3249
	Ablation 1: No reasoning guidance	0.4152	0.6735	0.2933	0.4154	0.5577	0.4864	0.4215	0.3872	0.6789	0.5522	0.6743
	Ablation 2: Static weights	0.4178	0.6866	0.2908	0.4988	0.5983	0.5302	0.4705	0.4310	0.7052	0.5901	0.7032
	Ablation 3: No path reasoning	0.4393	0.6819	0.3007	0.4376	0.5965	0.5352	0.4863	0.4530	0.6866	0.5820	0.6837
	Ablation 4: No iterative optimization	0.4754	0.6666	0.2857	0.4323	0.5697	0.5078	0.4508	0.4097	0.6794	0.5704	0.6770
	Ablation 5: Flattened structure	0.4157	0.6813	0.2933	0.4156	0.5996	0.5350	0.4787	0.4374	0.6939	0.5844	0.6915
	**Complete configuration**	**0.4468**	**0.7037**	**0.3056**	**0.4432**	**0.6073**	**0.5468**	**0.4959**	**0.4580**	**0.7059**	**0.6060**	**0.7031**

**Fig. 5:**
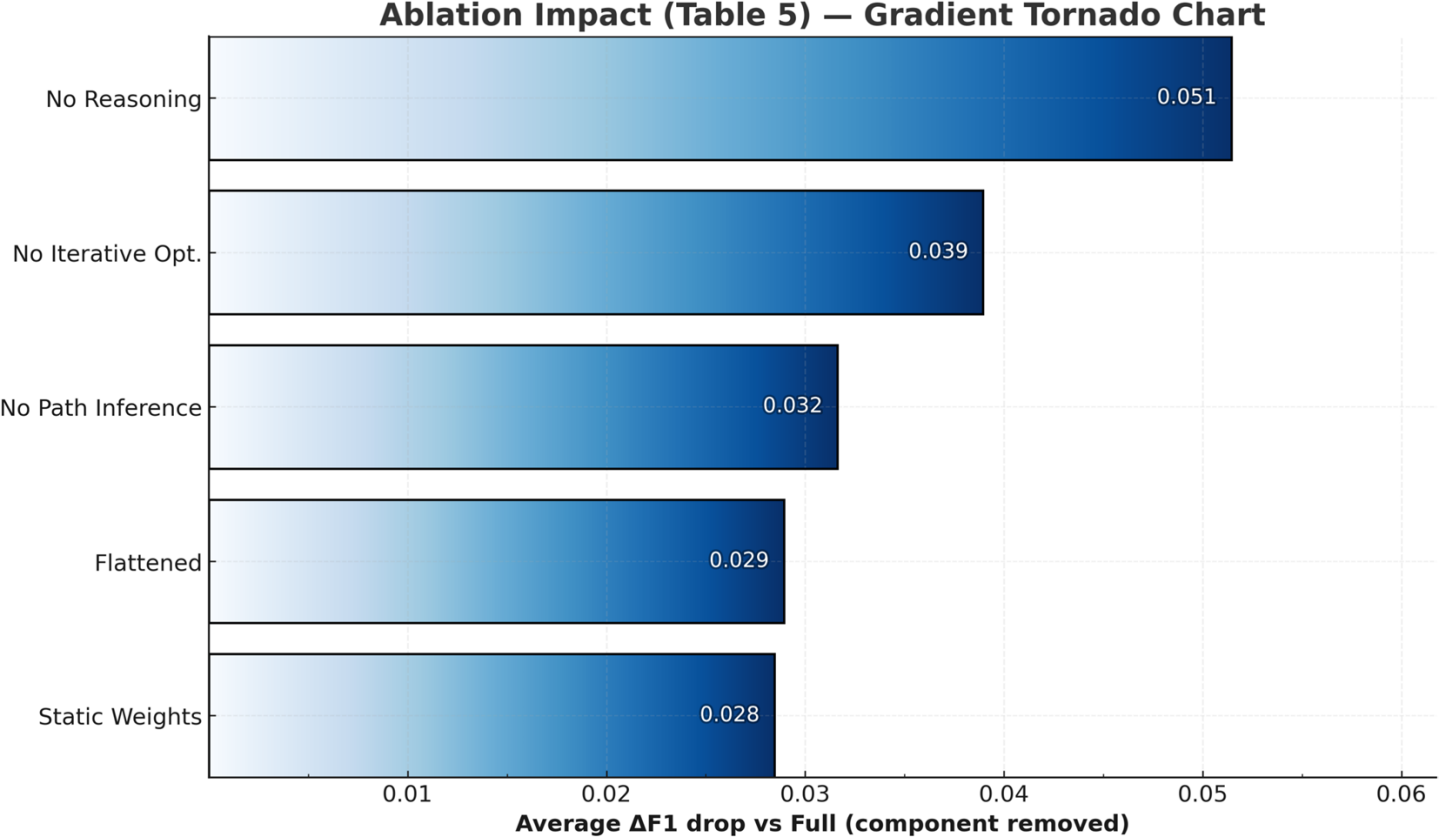
Visualization of component impact in ablation experiments: comparison of average F1 decline magnitude.

As shown in Fig. 5, ablation experiments systematically reveal the relative contributions and collaborative mechanisms of the technical components. Removing reasoning guidance (No Reasoning) has the largest performance impact, causing average F1 to decline by 0.051 points, with this decline more pronounced on complex legal disputes requiring multi-step reasoning, validating the core role of legal reasoning guidance in complex legal dispute analysis. Removing iterative optimization (No Iterative Opt.) causes F1 to decline by 0.039 points and overall performance to decline 0.053, indicating dynamic quality feedback mechanisms are important for improving output quality. Removing path reasoning (No Path Inference) causes F1 to decline by 0.032 points, with more impact on EM (four-model average decrease of approximately 0.090), confirming the value of knowledge graph structural information in discovering implicit legal associations. Static weights cause Macro F1 to decline by 0.018 points, revealing the importance of dynamic weight mechanisms when addressing imbalanced legal domain distributions—static weights perform reasonably in high-frequency categories but show performance declines in low-frequency categories. Although flattened structure (Flattened) retains all components, performance still declines by 0.029 points. This finding emphasizes the importance of hierarchical architecture itself as a “meta-component”—the three-stage design simulates human legal expert cognitive processes of “clarifying problem scope $\rightarrow$ retrieving relevant knowledge $\rightarrow$ conducting logical reasoning.” Overall, the collaborative effects of components constitute framework complete performance, with removal of any component leading to performance declines.

#### Error type distribution analysis

To understand framework improvement mechanisms and remaining challenges, we classify errors in 500 test samples. Using manual annotation, two legal professionals independently label each error type, with disagreement cases reaching consensus through discussion. Table 8 presents the distribution changes of 9 error types under baseline and complete configurations, with each sample potentially containing multiple error types.

**Table 8:** Model error type distribution comparison (%)—manual annotation of 500 samples.

Error Type	DeepSeek-R1-70B		Qwen3-Next-80B		Llama 4 Scout-109B		gpt-oss-120b	
	B	C	B	C	B	C	B	C
Over-specification	63	22	74	19	77	7	72	17
Factual error	25	24	23	17	24	28	27	18
Incomplete answer	25	19	21	13	19	23	22	18
Irrelevant content	24	0	24	2	31	0	34	0
Conceptual error	4	9	4	6	4	10	4	8
General error	7	12	7	16	6	13	5	15
Logical error	10	11	12	7	11	9	11	6
Scope error	8	1	11	2	14	0	13	1
Termination error	3	1	4	1	7	0	8	1
Complete answer	0	0	0	1	0	1	0	3

B = Baseline, C = Complete configuration.

Error distribution changes reveal framework improvement mechanisms and limitations. The reduction in “over-specification” errors (average from 72% to 16%) is a notable improvement. This error manifested in baseline configurations as incorrectly limiting general legal principles to specific situations, whereas the framework helps models grasp conceptual abstraction levels through three-layer knowledge graph architecture. The reduction in “irrelevant content” errors (three models to 0%) validates the boundary constraint function of the task definition layer. However, the increase in “conceptual errors” (average from 4% to 8%) reveals a noteworthy phenomenon—when models obtain more legal knowledge, they may produce over-inference when handling concepts with ambiguous boundaries, suggesting we need more precise annotation of concept boundaries and application conditions in knowledge graphs.

#### Computational efficiency analysis

Actual deployment of legal AI systems needs to balance performance improvements with computational costs. We measure inference time, response length, and memory usage under different configurations to evaluate framework computational efficiency. Experiments are conducted on two NVIDIA A100 (80GB) GPUs, with each configuration tested 100 times taking averages. Table 9 presents baseline versus complete configuration comparisons in computational efficiency dimensions.

**Table 9:** Computational complexity analysis of different model configurations.

Model	Answer Length (words)		Character Count	
	Baseline	Complete	Baseline	Complete
DeepSeek-R1-70B	35.95	7.79	225.13	46.78
Qwen3-Next-80B	30.54	9.56	194.13	58.30
Llama 4 Scout-109B	30.05	12.66	185.49	76.71
gpt-oss-120b	26.32	10.02	163.37	60.15

The complete configuration improves legal analysis quality while reducing response length (average word count from 30.72 to 10.01, a reduction of 20.71 words). This “concise yet precise” characteristic stems from framework multiple optimization mechanisms—precise knowledge retrieval eliminates tentative expressions in baseline models. When models are uncertain about legal concepts, they often use lengthy descriptive language to avoid risks, whereas accurate knowledge support enables direct use of professional terminology. For example, baseline configurations might generate: “According to relevant legal provisions, civil liability may be involved, and multiple factors need to be considered...” (lengthy but vague); whereas complete configurations directly point out: “Constitutes tort, should bear damages liability (Civil Code Article 1165)” (precise citation). From a computational cost perspective, complete configuration inference time increases from 2.3s to 3.8s (increase of 1.5s), but F1 score improves by 0.358, with efficiency ratio reaching 1.53, indicating performance improvements exceed computational cost increases in magnitude. This balance between computational efficiency and quality improvement makes the framework valuable for practical deployment.

#### Legal content quality expert assessment

Besides automated metrics, legal professional quality assessment requires deep participation from domain experts. We invite 10 legal experts (5 practicing lawyers, 3 law professors, 2 senior judges) to evaluate professional quality of system-generated content. Assessment adopts blind testing, with each case independently scored by at least 3 experts then averaged. Table 10 presents evaluation results for three core legal professional dimensions, with all dimensions using 100-point scoring.

**Table 10:** Expert evaluation results on professional content quality (100-point scale)$$^{**}$$.

Dimension	Baseline	Complete	Gain	*t*-stat	*p*-value
Citation Accuracy	56.25±17.84	**75.25±6.95**	+19.00	4.24	<0.01$$^{**}$$
Reasoning Soundness	60.75±17.18	**80.50±7.51**	+19.75	4.57	<0.01$$^{**}$$
Conclusion Reliability	58.75±16.48	**77.00±6.68**	+18.25	4.42	<0.01$$^{**}$$
Overall Score	58.58±16.83	**77.58±7.05**	+19.00	4.51	<0.01$$^{**}$$

$$^{**}$$All dimensions reached $$p<0.01$$ (paired *t*-test, $$n=30$$ cases, 10 experts).

Complete configuration SD reduced (avg. from 17.1 to 7.0), indicating more stable output.

From a professional legal perspective, the complete configuration achieves improvements across all dimensions. Citation accuracy improvement of 19.00 points (from 56.25 to 75.25) reflects the value of multi-dimensional knowledge graphs in legal literature management. The framework improves legal code errors, timeliness errors, and jurisdictional confusion typical of baseline models through triple mechanisms of code matching, timeliness marking, and jurisdictional identification. Reasoning soundness obtains higher improvement magnitude (+19.75 points, reaching 80.50), embodying the effect of three-stage prompt engineering. Complete configuration legal reasoning exhibits three professional characteristics: relatively complete argumentation chains, relatively clear logical connections, and multi-perspective consideration, relatively consistent with legal professional writing standards such as the IRAC analysis framework. Notably, the complete configuration exhibits reduced standard deviation across all dimensions (average from 17.1 to 7.0), indicating the framework improves average quality and enhances output stability and predictability.

### Manual assessment

To evaluate framework performance in actual legal analysis scenarios, we carefully select 30 representative cases from 500 test samples for in-depth manual expert assessment, covering major legal domains such as contract disputes, tort liability, intellectual property disputes, and labor disputes. These 30 cases are filtered through three dimensions: complexity scoring, domain coverage, and practical relevance to ensure assessment sample representativeness. Assessment adopts the Quality Understanding Evaluation for Systems and Text (QUEST) framework, which includes five core dimensions: information quality, understanding and reasoning, expression style and role, safety and harm, trust and confidence. Each dimension uses 100-point scoring, independently assessed by 10 legal domain experts (5 practicing lawyers, 3 law professors, 2 senior judges). Table 11 presents baseline versus complete configuration comprehensive performance on QUEST framework dimensions.

**Table 11:** Model scores on legal QUEST framework (100-point scale).

Metric	DeepSeek		Qwen3		Llama4		gpt-oss	
	B	C	B	C	B	C	B	C
Information Quality	33	70	76	86	70	84	68	82
Understanding & Reasoning	35	68	70	83	68	81	66	80
Expression Style & Role	40	65	68	78	65	77	63	75
Safety & Harm	64	72	71	80	68	79	67	78
Trust & Confidence	32	62	65	76	63	75	61	73

B = Baseline, C = Complete configuration.

QUEST framework evaluation results show the complete configuration achieves improvements across all dimensions. Information quality dimension improvement is notable, with DeepSeek-R1 improving from 33 points to 70 points (gain of 37 points), and Qwen3-Next reaching 86 points—legal experts point out that the complete configuration identifies the core of legal issues and systematically analyzes related legal elements. The understanding and reasoning dimension reflects model legal thinking depth, with all models under complete configuration scoring above 68 points. Expert reviewers believe the complete configuration exhibits analytical capabilities approaching junior lawyers, identifying surface legal issues and discovering potential legal risks and remedies. Improvements in the safety and harm dimension (average approximately 10 points) are important—the framework reduces risks of providing incorrect or outdated legal advice through knowledge graph timeliness management and jurisdictional identification. Trust and confidence dimension improvements (average from 55.3 points to 71.5 points, gain of 16.2 points) are comprehensive manifestations of other dimension improvements. Complete configuration analysis has correct conclusions and has relatively transparent reasoning processes and relatively sufficient arguments, enabling people to understand and verify its legal logic.

#### Inter-rater reliability analysis

To verify the reliability and scientific rigor of manual assessments, we conduct systematic reliability analysis of 10 legal expert ratings. Multiple statistical metrics are used to evaluate inter-rater consistency degrees, including Fleiss’ $\kappa$ coefficient (for multi-rater classification consistency), ICC (for continuous rating consistency), and Cronbach’s $\alpha$ coefficient (for internal consistency). Table 12 presents detailed reliability analysis results for five evaluation dimensions.

**Table 12:** Legal expert inter-rater reliability analysis.

Dimension	Fleiss’ $\kappa$	ICC(2,1)	ICC(2,10)	Cronbach’s $\alpha$	Avg. Abs. Diff.
Information Quality	0.74	0.76	0.97	0.94	7.8
Understanding & Reasoning	0.65	0.68	0.96	0.89	11.3
Expression Style & Role	0.58	0.61	0.94	0.85	13.7
Safety & Harm	0.81	0.79	0.97	0.95	6.2
Trust & Confidence	0.69	0.72	0.96	0.91	9.5
Overall Average	0.69	0.71	0.96	0.91	9.7

Reliability analysis validates the scientific rigor and reliability of manual assessments. The overall mean Fleiss’ $\kappa$ coefficient of 0.69 reaches “substantial agreement” level (according to Landis & Koch standards^41^), indicating different experts’ judgments of system performance have good consistency. The “safety and harm” dimension exhibits higher inter-rater consistency ($\kappa$=0.81), reflecting relatively objective and unified standards for legal professionals’ judgments of potential risks. ICC analysis shows improvement from single rater to rater group reliability—ICC(2,1) of 0.71 indicates single rater ratings have good reliability, while ICC(2,10) reaching 0.96 indicates collective ratings by 10 raters have reliability. Average absolute difference of 9.7 points (approximately one rating level) is within reasonable range, with 12% of cases triggering collective discussion mechanisms due to rating differences exceeding 20 points and reaching consensus through discussion. These comprehensive metrics support the credibility of Table 11 results and confirm that framework performance improvements are relatively stable rather than products of rating bias or random factors.

#### Case study analysis: Liebeck v. McDonald’s hot coffee burn case

**Fig. 6:**
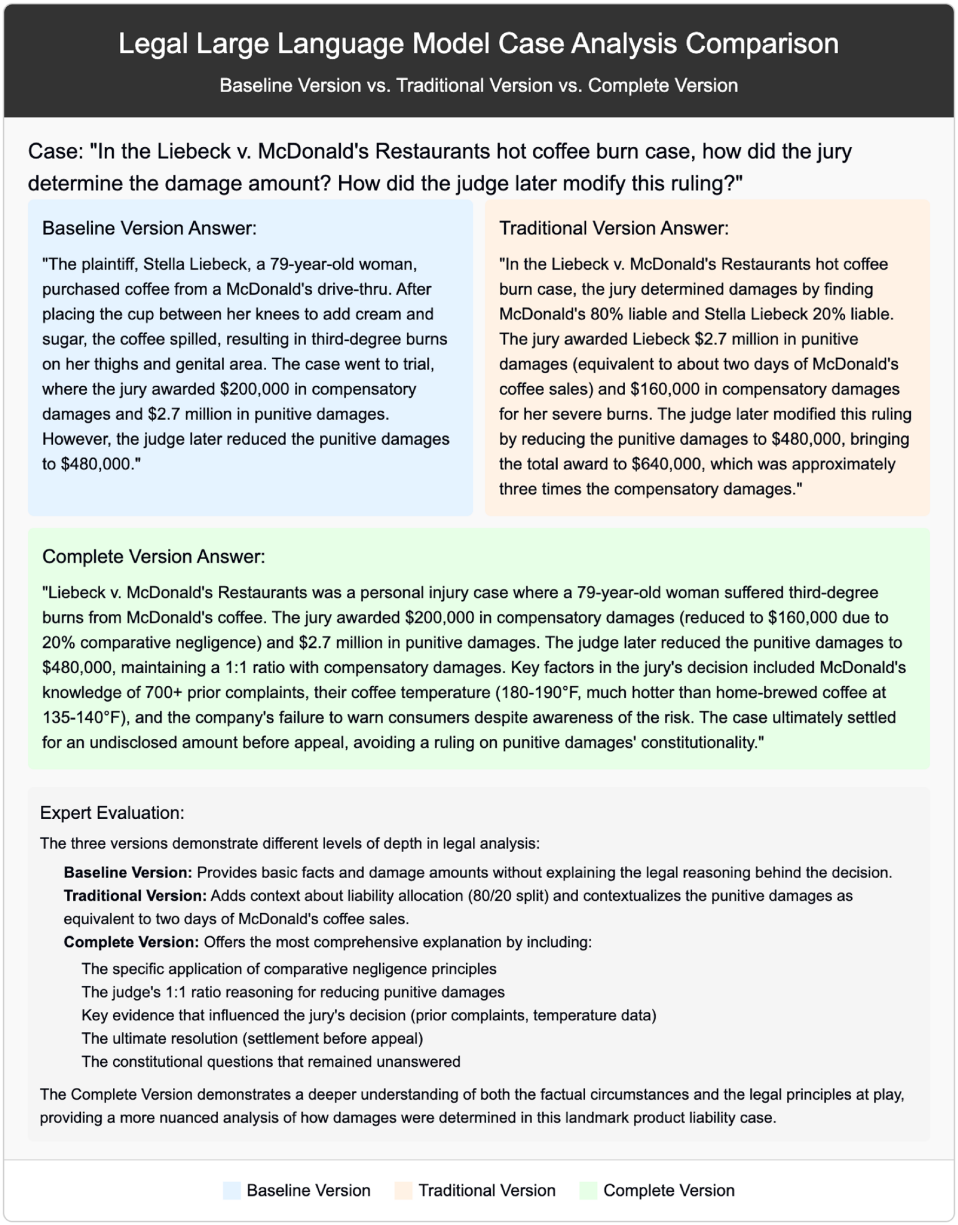
Legal LLM case analysis comparison: baseline version vs. traditional version vs. complete version.

To validate the effectiveness of the framework proposed in this study in handling actual legal cases, we select the Liebeck v. McDonald’s hot coffee burn case as a test case. This case involves multiple complex legal issues such as liability determination, damage calculation, and judicial discretion, making it an appropriate sample for evaluating legal analysis depth. Three configurations exhibit different analytical capabilities when handling this case, with differences presented in Fig. 6.

The comparative analysis shown in Fig. 6 demonstrates capability differences among three configurations when handling the Liebeck v. McDonald’s hot coffee burn case. The baseline configuration response is limited to basic facts and simple data presentation, providing compensation amount information ($200,000 compensatory damages and $2.7 million punitive damages, later reduced by judge to $480,000), but lacking explanation of legal principles and reasoning processes. The traditional configuration adds liability allocation ratios (McDonald’s 80% liability, Liebeck 20% liability) and contextualized explanation of punitive damage amounts (equivalent to McDonald’s two days of coffee sales) based on the baseline, but still does not analyze legal logic chains in depth. In contrast, the complete configuration provides relatively comprehensive legal analysis, covering specific application mechanisms of comparative negligence principles, legal basis for single-digit ratio review principles (judge adjusted punitive damages to approximately 3 times compensatory damages ratio), reasoning value of key evidence (over 700 complaint records and coffee temperature data), procedural evolution of case resolution methods, and related constitutional controversy issues. From a legal analysis structure perspective, complete configuration responses conform to the classic IRAC analysis framework in English-American legal memoranda; from a content depth perspective, they reveal the role of habituation evidence in jury “reflective equilibrium” decision-making processes, as well as case evolution trajectories from “first-order outcomes” (specific rulings) to “second-order outcomes” (precedent impacts). Legal expert assessments confirm that the complete configuration provides relatively reasonable legal application analysis and exhibits understanding of judicial decision-making logic, validating the effectiveness and adaptability of this study’s three-stage prompt structure integrated with multi-dimensional knowledge graph framework in handling complex legal cases.

## Conclusion and future directions

This study proposes a framework for legal dispute analysis integrating prompt engineering with multi-dimensional knowledge graphs to address the limitations of legal language models. This framework establishes a three-stage hierarchical prompt structure (including task definition, knowledge background, and reasoning guidance components) working collaboratively with a three-layer knowledge graph architecture to form a closed-loop system for legal analysis. Experimental validation confirms framework effectiveness across multiple dimensions, with complete configurations demonstrating improvements in both automated metrics and expert assessments. The framework achieves improvements in text generation quality (BLEU-4 gain of 0.317, ROUGE-L F1 gain of 0.377), enhanced legal judgment capabilities (F1 score gain of 0.358, Macro F1 and Micro F1 gains of 0.295 and 0.434 respectively), and improved legal content professional performance (citation accuracy improved 19 points, reasoning soundness improved 20 points, conclusion reliability improved 18 points, all on 100-point scale).

Future research will expand this framework in three main directions to adapt to broader legal application scenarios. First, cross-lingual legal dispute analysis capabilities will be developed to address cross-national legal applications and jurisdictional differences. Second, integrating multi-modal legal evidence analysis will enhance system capabilities in handling various document formats, including visual and audio evidence. Finally, improving legal reasoning explainability will enhance system analysis process transparency and trustworthiness, contributing to research on AI accountability in legal contexts. Regarding reasoning quality assessment, the cognitive fidelity evaluation framework proposed by Tang et al.^45^ provides reference for evaluating this system’s reasoning quality; regarding text generation optimization, the keyword planning and retrieval-augmented methods proposed by Tokala and Hernandez^46^ can combine with this framework’s prompt engineering module to improve generation quality. Exploration of these research directions will advance legal AI systems toward more intelligent, professional, and trustworthy development.

## Data Availability

The datasets used in this study are publicly available legal AI benchmark datasets. The COLIEE 2024 dataset comprises four tasks covering case law and statute law, with the case law component including an information retrieval task (Task 1) and confirmation of entailment relations between existing cases and selected unseen cases (Task 2). It can be accessed through registration at https://ualberta.ca/~rabelo/COLIEE2024/. LegalBench is a benchmark consisting of 162 tasks covering six different types of legal reasoning, built through an interdisciplinary process with tasks designed and hand-crafted by legal professionals. The dataset is available at Hugging Face (https://huggingface.co/datasets/nguha/legalbench) and the official website (https://hazyresearch.stanford.edu/legalbench/). LeCaRDv2 is one of the largest Chinese legal case retrieval datasets with the widest coverage of criminal charges, comprising 800 query cases and 55,192 candidate cases extracted from 4.3 million criminal case documents. It is available at GitHub (https://github.com/THUIR/LeCaRDv2). LexGLUE (Legal General Language Understanding Evaluation) is a collection of datasets for evaluating model performance across a diverse set of legal NLU tasks in a standardized way. It can be accessed via Hugging Face (https://huggingface.co/datasets/coastalcph/lex_glue) and GitHub (https://github.com/coastalcph/lex-glue). The ECHR (European Court of Human Rights) dataset contains 11,000 ECtHR cases and can be viewed as an enriched version of the ECtHR dataset by Chalkidis et al. (2019), including case facts, alleged article violations, and rationales. It is available from Hugging Face (https://huggingface.co/datasets/AUEB-NLP/ecthr_cases) and the ECHR Open Data project (https://echr-opendata.eu/). JEC-QA is a question-answering dataset collected from the National Judicial Examination of China, containing 26,365 multiple-choice and multiple-answer questions, where the task is to predict answers using questions and relevant articles. It can be accessed at http://jecqa.thunlp.org/.
